# CrystalTac: Vision-Based Tactile Sensor Family Fabricated via Rapid Monolithic Manufacturing

**DOI:** 10.34133/cbsystems.0231

**Published:** 2025-04-10

**Authors:** Wen Fan, Haoran Li, Dandan Zhang

**Affiliations:** ^1^ Department of Bioengineering, Imperial College London, London, UK.; ^2^ Bristol Robotics Lab, School of Engineering Mathematics and Technology, University of Bristol, Bristol, UK.

## Abstract

Recently, vision-based tactile sensors (VBTSs) have gained popularity in robotics systems. The sensing mechanisms of most VBTSs can be categorized based on the type of tactile features they capture. Each category requires specific structural designs to convert physical contact into optical information. The complex architectures of VBTSs pose challenges for traditional manufacturing techniques in terms of design flexibility, cost-effectiveness, and quality stability. Previous research has shown that monolithic manufacturing using multimaterial 3-dimensional printing technology can address these challenges but fails to bridge the gap between the design phase and creation phase of VBTSs. Thereby, in this study, we introduce the CrystalTac family, a series of VBTSs designed with on-demand sensing mechanisms and fabricated through rapid monolithic manufacturing. Case studies on the CrystalTac family demonstrate their efficiency in targeted tasks involving tactile perception, along with impressive cost-effectiveness and design flexibility. The CrystalTac family aims to highlight the potential of rapid monolithic manufacturing techniques in VBTS development and inspire further research in tactile sensing and manipulation.

## Introduction

The integration of tactile sensors with robots has drawn considerable interest in fields ranging from soft robotics [1] and bionic robotics [2] to human–robot interface areas [3]. Among the different types of tactile sensors [4–6], vision-based tactile sensors (VBTSs) [7,8] use cameras to record the physical deformation when interacting with external objects. This method has gained increasing attention due to its superior spatial resolution on tactile sensing and relatively simple structure. The rapid adoption of VBTSs is largely due to advancements in computer vision. Vision sensors, such as RGB cameras, project a 3-dimensional (3D) scene into 2-dimensional (2D) frames, converting the color, shape, and motion information of the external environment into a distribution of pixel values. In comparison, VBTSs operate similarly to reprographic devices, scanning and mapping 2.5-dimensional (2.5D) features [9] into 2D images. These features, such as the texture geometry of the contact surface or shear force around the touched area, are termed 2.5D because their perception depth is confined to a limited range and cannot be extended arbitrarily along the direction of light projection as 3D visual features can. Due to this limitation, VBTSs need a specific medium to convert physical information into optical signals that the camera can detect. The realization of such a medium generally involves 4 steps: (a) establishing the correlation between tactile and optical features, often known as the tactile sensing mechanism; (b) creating the sensor architecture to embody this tactile sensing mechanism; (c) choosing appropriate techniques to manufacture the sensor’s sub-components; and (d) finalizing the entire hardware assembly process based on the produced sub-components. In conclusion, the initial 2 steps fall under the “design” phase of VBTSs, while the final 2 steps comprise the “creation” phase. The design phase frequently imposes difficulties on the creation phase. This is due to the conventional method’s segregation of design, manufacturing, and assembly stages.

Among the 3 primary modules in VBTSs [8], the illumination and vision modules are typically procured from commercial electronic component suppliers. In contrast, the contact module presents the toughest challenge [10], thereby the subsequent discussion of VBTS manufacturing primarily refers to the contact module. A VBTS contact module normally consists of 6 main components: a base, a lens, an elastomer, a marker pattern, a coating, and a skin, which are often produced using diverse manufacturing techniques. As analyzed by Fan et al. [11], the main challenges in VBTS manufacturing include complexities in design, process, time, and quality, which impact the design and creation of VBTSs.

There have been several research studies focused on the design and creation of VBTSs [10–14]. Beyond the mold-casting and split-printing methods of elastomer manufacturing, the integral printing principle [10] is established to enable a fully 3D-printed VBTS, MagicTac. Based on integral printing, monolithic manufacturing technology [11] is proposed to fabricate the C-Sight tactile sensor with a more complicated structure, which highlights advancements in multimaterial 3D printing. By simplifying VBTS manufacturing into a single printing sequence, this previous work accelerates VBTS production and ensures greater consistency and integration of the sub-components. It is expected to substantially improve the reliability, productivity, and affordability of the newly developed tactile sensor. However, previous work is limited to fabricating a design that embodies a fixed kind of sensing mechanism, lacking a general framework on guiding how to design various VBTSs utilizing monolithic manufacturing. In other words, the previous work solves only the problem of “how to make an existing VBTS design through monolithic manufacturing” but is still limited to another problem of “what kind of VBTSs can be designed for monolithic manufacturing”. Therefore, the gap between the design and creation of VBTSs should be bridged more efficiently, leading to rapid monolithic manufacturing.

To better explore the potential of this technology and solve the challenge of “what kind of VBTSs can be designed for monolithic manufacturing”, we summarize mainstream VBTSs into 5 categories based on their sensing mechanisms and then propose to develop a CrystalTac-type sensor following this categorization, also known as the CrystalTac family, aiming to demonstrate the versatility of monolithic manufacturing and minimize the gap between VBTS design and creation. The name “CrystalTac” draws on the properties of the crystal, a mineral known for its clarity and variability in color and texture when combined with other minerals, just like the versatile structure of the different tactile-sensing-mechanism-based VBTSs. The rapid monolithic manufacturing technology should be competent for the fabrication of the CrystalTac family by adapting to different design needs, including customized sensing principles, overall dimensions, and architectural details.

The main contributions of this paper are listed as follows:

• We summarized the design and creation methods of known VBTSs and proposed a new categorization method to encapsulate their typical sensing mechanisms, including the intensity mapping method (IMM), the marker displacement method (MDM), the modality fusion method (MFM), and multimechanism fusion.
• We investigated the manufacturing feasibility of VBTSs based on different sensing mechanisms produced through the monolithic manufacturing method in terms of technological and practical feasibility, bridging the gap between design and creation of VBTSs.
• We developed the CrystalTac family, a series of sensors that includes C-Tac, C-Sight, C-SighTac, Vi-C-Tac, and Vi-C-Sight, each design based on a unique tactile sensing mechanism, aiming to demonstrate design-led flexible creation through rapid monolithic manufacturing.
• We conducted functional experiments to evaluate the sensing performance, cost-effectiveness, and design flexibility of CrystalTac-type sensors. Also, optimization upon sub-component manufacturing and several novel marker designs are introduced.

## Materials

Based on the 4 key steps for realizing the “design” and “creation” of VBTSs introduced in the previous section, we review most of the known VBTSs and summarize their sensing mechanisms, manufacturing processes, and assembly methods.

### Typical sensing mechanism of known VBTSs

**Fig. 1.**
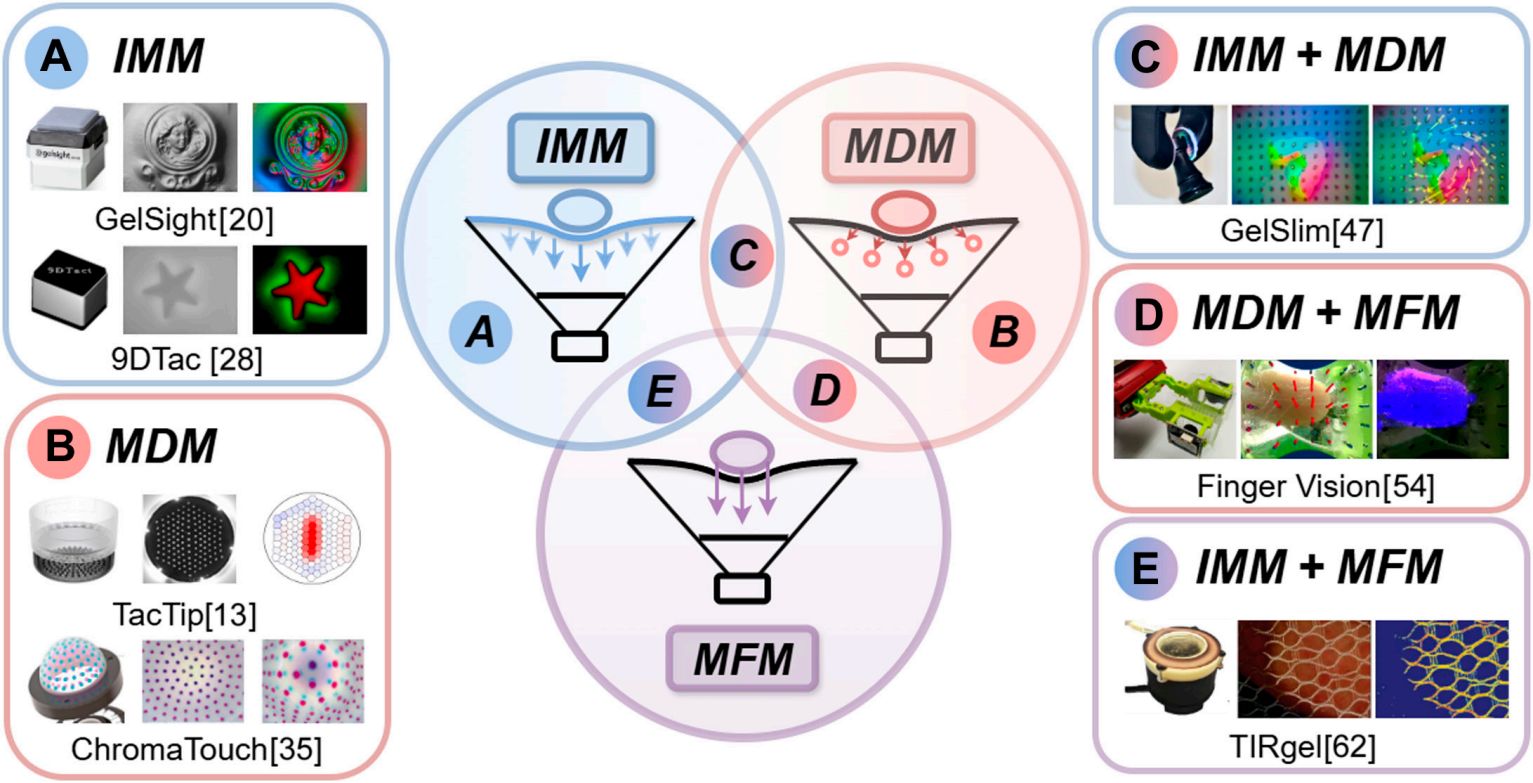
Typical tactile sensing mechanisms of known vision-based tactile sensors (VBTSs). (A) Intensity mapping method (IMM), such as GelSight and 9DTac [20,28]. (B) Marker displacement method (MDM), such as TacTip and ChromaTouch [13,78]. (C to E) Multimechanism fusion consisting of IMM + MDM, MDM + modality fusion method (MFM), and IMM + MFM, such as GelSlim, Finger Vision, and TIRgel [47,62,79].

As shown in Fig. 1, the sensing mechanisms of most VBTSs [8,15] can be categorized into several distinct methods: IMM, MDM, MFM, and multimechanism fusion.

#### Intensity mapping method

IMM uses the pixel intensity values from the imaging frames to indicate tactile features, as illustrated in Fig. 1A. This approach leverages the high resolution of the VBTS camera frame and the changes in each pixel value over a threshold interval to reconstruct continuous features, including fine geometric textures and dense motion distributions. This change in pixel value requires the VBTS to incorporate specific structures, such as a light-conductive plate or a reflective coating. For example, the earlier optical waveguide-type sensors [7,16–18] usually rely on total internal reflection (TIR) [19] to map the contact pattern located on the light-conductive plate surface. GelSight-type sensors [20], such as OmniTact [21], Digit [22], GelTip [23], InSight [24], and Tactile Fingertip [25], employ a coating layer with uniform reflective properties applied to a silicone elastomer, enabling pixel-level tactile sensing. By incorporating RGB lighting, GelSight captures multiple images under different lighting conditions within a single frame, facilitating the estimation of the overconstrained gradient of the contact surface geometry through the photometric stereo algorithm [9,26]. In contrast to intensity mapping via a light-conductive plate or a reflective coating, DTac-type sensors [27], such as 9DTac [28], employ a combination of translucent gel and an opaque layer, where variations in pixel darkness can indicate intensity-to-depth regressions or even estimate force/torque. If a depth camera is used instead of a traditional RGB camera in the VBTS, each pixel intensity value will also include real physical distance information in addition to color information. Li et al. [29] integrated a latex inflatable film with a depth camera, achieving precise deformation measurements through stereovision. Furthermore, with the progress of materials science, mechanoluminescent materials [30] and mechanoresponsive color-changing materials [31] have been used for VBTS design that can emit photonic signals or change their color pattern in response to touch and deformation; thereby, their pixel intensity of chromatic distribution also can map tactile features.

#### Marker displacement method

MDM, depicted in Fig. 1B, translates tactile information into displacement distributions of marker patterns either positioned on the surface or embedded within the elastomer. In the paper by Li et al. [32], MDM is classified into 2D MDM, 2.5D MDM, and 3D MDM, based on the type of tactile features represented by the pattern changes. Both 2D MDM and 2.5D MDM employ a single camera to capture the 2D movements of markers in horizontal and vertical directions. However, 2.5D MDM can indirectly represent corresponding contact depth information by observing the deformable characteristics of the markers, such as changes in size or shape. In contrast, 3D MDM precisely captures the depth of markers using stereovision. GelForce [33,34] uses 2 layers of spherical marker arrays to measure 3D force distributions. Inspired by the dermal papillae in the human fingertip, TacTip [35–37] designs pin-shaped markers, which amplify contact information through leverage principles. Furthermore, Yang et al. [38] employed marker displacements to estimate the gradient of the contact surface through the integration method. ChromaTouch [13] introduces 2 superimposed color filters as a composite marker pattern, where contact deformation alters the markers’ hue, centroid, and apparent size. Similar designs are found in the studies by Lin and Wiertlewski [39] and Lin et al. [40]. DelTact [41], Viko [42], and Du et al.’s sensor [43] all utilize a dense random color pattern for precise tracking of contact deformations. Sferrazza and D’Andrea [44] randomly embedded spherical fluorescent green markers within an elastomer to enhance the sensing range. A multicolor continuous marker pattern [45] has been developed to improve the representation and extraction of VBTS contact information. Tac3D [46] employs virtual binocular vision to precisely measure the 3D shape and force distribution of the contact surface using a continuous marker pattern.

#### Modality fusion method

MFM integrates multimodal features beyond sole tactile input, typically incorporating vision, proximity, and temperature sensing. This integration mitigates the inherent limitations of a single tactile modality, such as the inability to perceive features like color, distance, and temperature. However, MFM cannot operate independently in VBTSs and needs to be combined with IMM or MDM for tactile sensing, thereby included in the concept of multimechanism fusion.

#### Multimechanism fusion

The sensing mechanisms for VBTSs introduced above each offer distinct advantages. However, researchers have been proactively developing multimechanism fusion sensors to further improve the performance of VBTSs by obtaining comprehensive sensing information.

• IMM + MDM: GelSight-type sensors [20] incorporate dot markers above the reflective coating layer, aiming for force and shear detection rather than solely capturing fine textures, as demonstrated by GelSlim [47], shown in Fig. 1C; GelSight Wedge [48]; and GelStereo [49]. DenseTact [50] employs a randomized dense pattern with a coating to extract continuous tactile features. The Soft-bubble sensor [51] utilizes a stereo camera for the tracking of shear-induced displacement through custom marker patterns. Wang et al. [52] introduced particle image velocimetry to establish a linear relationship between force and velocity. UVtac [53] employs a switchable ultraviolet (UV) method to decouple marker patterns and reflective membrane images, facilitating both object localization and force estimation, with each functionality remaining independent of the other.
• MDM + MFM: Finger Vision [54], as illustrated in Fig. 1D, embeds black marker patterns on a transparent elastomer to achieve multimodality sensing. The approach proposed by Yamaguchi [55] increases the force resolution and sensitivity of Finger Vision by introducing whiskers. ViTacTip [56] also employs a transparent skin to enhance TacTip with a vision–tactile fusion capability. Similar to UVtac, SpecTac [57] uses UV light-emitting diodes and randomly distributed UV fluorescent markers, allowing for a switch between visual and tactile sensing modes, controlled by the activation of the UV light-emitting diodes. SATac [58] introduces a thermoluminescence layer to capture temperature modality, combined with a marker array for pressure and shear sensing.
• IMM + MFM: A light-conductive plate and compound-eye camera was employed by Shimonomura et al. [7] to capture both tactile and proximity information. The tactile feature is obtained as an infrared image through TIR, while the proximity feature is detected through stereo matching. See-Through-your-Skin [59] utilizes a translucent coating and adjustable lighting to enable the transition between visual and tactile modalities. In contrast, StereoTac [60] and VisTac [61] incorporate binocular cameras, extending 2D visual sensing into 3D areas. Shown in Fig. 1E, TIRgel [62] uses TIR to visualize tactile features, achieving conversion between modalities via focus adjustment. M^3^Tac introduces near- and mid-infrared cameras to capture multispectral imaging for multimodality sensing, including texture, force, and temperature.

### Typical manufacturing method of known VBTSs

Based on Table 1, the manufacturing methods of sub-components in known VBTSs are discussed below. The different tactile sensing mechanisms lead to variations in their manufacturing processes, where multimechanism fusion further complicates the procedure.

**Table 1.** Comparison of typical VBTSs in terms of tactile sensing mechanism and manufacturing method. “-” indicates that the sub-part is not included. * stands for the sensors by Zhang et al. [65] and Solyaman et al. [69]. Boldface indicates the only 3 VBTSs that fabricate the marker or skin through the 3D-printing method, while all others rely on the traditional way.

Sensor	Mechanism	Base	Lens	Elastomer	Marker	Skin	Coating
Digit [22]	IMM	Mold-formed	Laser-cut	Mold-formed	-	-	Airbrushed
OmniTact [21]	IMM	3D-printed	-	Mold-formed	-	-	Hand-painted
9DTac [28]	IMM	3D-printed	Laser-cut	Mold-formed	-	Mold-formed	-
* [65]	IMM	3D-printed	Laser-cut	DIY-modified	-	-	Hand-gilded
TacTip [35–37]	MDM	**3D-printed**	Laser-cut	Injection-filled	**3D-printed**	**3D-printed**	-
ChromaTouch [13]	MDM	**3D-printed**	-	Injection-filled	**3D-printed**	**3D-printed**	-
DelTact [41]	MDM	3D-printed	Laser-cut	Mold-formed	Film-sticked	Airbrushed	-
* [69]	MDM	**3D-printed**	-	-	Pad-printed	**3D-printed**	-
GelSlim 3.0 [47]	IMM + MDM	3D-printed	Commercially ordered	Mold-formed	Ink-printed	Airbrushed	Airbrushed
GelSight Wedge [48]	IMM + MDM	3D-printed	Laser-cut	Mold-formed	Ink-printed	Film-sticked	Airbrushed
DenseTact 2.0 [50]	IMM + MDM	3D-printed	Laser-cut	Mold-formed	Ink-printed	-	Paint-dipping
UVtac [53]	IMM + MDM	3D-printed	Laser-cut	Mold-formed	Ink-printed	-	Airbrushed
Finger Vision [55]	MDM + MFM	3D-printed	Laser-cut	Mold-formed	Solid-embedded	Film-sticked	-
ViTacTip [56]	MDM + MFM	**3D-printed**	Laser-cut	Injected-filled	**3D-printed**	**3D-printed**	-
SpecTac [57]	MDM + MFM	3D-printed	Laser-cut	Mold-formed	Hand-painted	Hand-painted	-
SATac [58]	MDM + MFM	3D-printed	Laser-cut	Mold-formed	Laser-cut	Knife-coated	Knife-coated
STS [59]	IMM + MFM	3D-printed	Laser-cut	Mold-formed	-	Airbrushed	Airbrushed
VisTac [61]	IMM + MFM	3D-printed	Laser-cut	Mold-formed	-	Hand-painted	Airbrushed
TIRgel [62]	IMM + MFM	3D-printed	Laser-cut	Mold-formed	-	-	-
M^3^Tac [72]	IMM + MFM	3D-printed	-	Commercially ordered	-	-	Commercially ordered

3D, 3-dimensionally; DIY, do-it-yourself; STS, See-Through-your-Skin

#### Base

The base, also known as the case, mount, frame, body, bracket, or housing, typically serves as the external structure of VBTSs. It acts as the connecting element between the contact module and the illumination/vision modules. To ensure a secure fit among these modules and to achieve the required durability and compactness of the sensors, the base must be designed with an appropriate shape and constructed from a material of sufficient rigidity. Three-dimensional printing technology [20,21,25,28,38,42,43,50,51,53,62,63] is the most common option for prototyping, while commercially large-scale productions usually rely on the mold-forming method [22].

#### Lens

The lens allows the camera to capture tactile information without obstruction. Additionally, it internally supports the elastomer during interactions. The common manufacturing method employs a laser cutter to shape the acrylic board [20,27,34,41,42,51,54,60–63]. However, lenses with complex curved surfaces, diverging from simple planar shapes, can only be produced through mold-forming methods [25] or procured from commercial suppliers [47]. Additionally, some sensors [13,21,24] do not incorporate lenses but rely on their inherent structure or an internal skeleton.

#### Elastomer

The elastomer serves as one of the primary mediums for converting tactile information into visual data, with core properties including transparency, color, and hardness. Mold-formed silicone [12,64] is the material most preferred for creating these elastomers, as it offers adjustable properties to suit various applications [13,14,20,38,41–44,47,50,53,54,59–62]. The preparation process involves A/B solution mixing, mold casting, bubble removal, and heat curing. Translucent and colored silicone may require dyeing with a pigment [28]. The adjustable hardness depends on the variation of the ratio of mixed solutions [25]. Some VBTSs use commercial products as alternatives, modified using do-it-yourself (DIY) methods [65,66], to simplify manufacturing. There are exceptions, such as Soft-bubble [51], which features an air-filled membrane design, while TacTip [36], BioTacTip [63], and Ito et al.’s sensor [67] use mixed ultrasoft gel (Techsil RTA27905 A/B) and colored water, respectively.

#### Marker

For MDM-type VBTSs, markers play the role of mapping tactile deformation to visual pattern distribution. In the paper by Li et al. [32], different marker patterns are categorized into 2D, 2.5D, and 3D types, posing huge challenges to manufacturing. Referring to Zhang et al. [12], we classify the marker manufacturing methods into 3 types based on the location where markers are fabricated:

• Surface fabrication: Markers are prepared on the surface of premade elastomers through direct or indirect methods. The former approach combines light etching, mask templates, and stamp plates with ink printing [47,50,52,53,58,68] or utilizes plastic beads [38,54]. Similarly, SpecTac [57,66] manually apply fluorescent markers using a brush and a UV pen, respectively. In the latter case, the complete marker patterns are printed in advance using materials such as sticker film [43], transfer paper film [49], pad printing [69], or adhesive-backed templates [51] and then applied to the surface of the elastomer.
• Embedded fabrication: To capture the deformation field at different depths, rather than solely at the contact plane, markers are embedded within the elastomer. Sferrazza and D’Andrea [44] incorporated fluorescent markers into the solutions during the preparation of the silicone. GelForce [33,34] and Lin and Wiertlewski’s [39] and Lin et al.’s sensor [40] had 2 marker arrays of different colors placed in the elastomer, prepared layer by layer.
• Integral fabrication: Markers are prepared integrally with the skin in a single piece. Pin-shaped markers with the skin of TacTip were initially produced by mold casting [70]. Subsequently, 3D printing has been introduced for the later versions of MDM-type VBTSs [36,37,56,63].

#### Skin

The skin serves as the direct contact interface with the external environment. For VBTSs designed with a silicone elastomer as the main body, some lack a skin layer [20,21,41,53,54,62,65], while others employ a skin for protection, which is achieved by casting a thin layer of silicone through mold forming [38,61] or spray painting [53,59,60]. To filter noise, DTac [27,28] has black silicone manually applied on the top surface, similarly to sensors by Sato et al. [34], Du et al. [43], and Sferrazza and D’Andrea [44]. Several designs implement fabric films or adhesive tape films as a skin-like layer, as demonstrated by Zhang et al. [46], Wang et al. [48], and Ma et al. [71]. For example, Finger Vision [54] employs a thin transparent plastic film to protect the silicone body from contaminants. Some VBTSs rely on the skin as the primary structural component.

TacTip [35–37] and ChromaTouch [13] and Solayman et al.’s sensor [69] have the skin built as the main body through a 3D-printing method with PolyJet and direct ink writing, respectively. In comparison, PolyJet has advantages in multimaterial printing ability, print efficiency, and print quality. ChromaTouch also requires the casting of a white silicone layer to filter noise, while Solayman et al.’s sensor [69] calls for an extra marker-adding process through pad printing. As evaluated by Solayman et al. [69], the mechanical behavior of the 3D-printed skin proved to be sufficient to meet the needs of frequent contact involving force and shear in real robotics tasks like mold casting. Furthermore, Soft-bubble [51], M^3^Tac [72], and Li et al.’s sensor [29] use hand-cut or commercially ordered latex films as outer skin to form an air-filled structure.

#### Coating

Coating layers are widely used in IMM VBTSs for fine tactile feature mapping. To make a distinction from the skin, the coating is generally defined as a thin, functional layer that is more fragile and prefers to be wrapped inside the skin without direct contact with the external environment. However, both as the outer layer of VBTS, the skin and coating may overlap in function; for example, some coating layers can also filter noise and keep moving toward better abrasion resistance. The coating can be categorized into 2 broad types: reflective coatings and controllable coatings.

• Reflective coating: GelSight-type sensors [20] incorporate metal pigments into silicone, using bronze flakes for semispecular coatings and fine aluminum powder for matte coatings. Various manufacturing methods are employed, such as brush painting [21], airbrushing [22,47,66], sputtering [73], and the paint dipping technique [50]. Similarly, Zhang et al. [65] used metal foil to create a semimirror coating through the gilding process.
• Controllable coating: Some MFM-based VBTSs control the coating transparency by adjusting internal lighting conditions. See-Through-your-Skin [59], StereoTac [60], and VisTac [61] all apply 2 to 3 layers of “mirror spray” to achieve a translucent layer as the controllable coating. M^3^Tac [72] uses a commercially customized silver film upon latex to achieve a similar function.

### Typical assembly method of known VBTSs

Compared to VBTS design and manufacturing processes, the assembly strategy is often overlooked in most of the current research, which is usually mixed in the manufacturing process. However, based on our actual production experience, the above neglect of the proper assembly strategy will cause a series of problems, such as the amplification of sub-part manufacturing errors, resulting in the life, performance, and reproducibility of the sensor varying obviously. Some researchers have taken note of this problem, such as GelSlim 4.0 [74], but this is still limited to skill-intensive hand assembly. The suitable assembly strategy should be efficient and cost-effective, with a high degree of consistency in the quality of the final product. Therefore, it is necessary to explore and summarize the assembly of the typical VBTS. Here, the assembly process is analyzed across 3 dimensions: tools, workflows, and mechanisms.

#### Assembly tool

The assembly tool consists of manual assembly and machine assembly. Due to the widespread use of traditional manufacturing methods, such as mold forming [20,55], manual assembly remains the prevalent practice. Machine assembly, characterized by the use of specific devices such as multimaterial 3D printers, is exemplified by MagicTac [10] and C-Sight [11]. Generally, manual assembly may result in unpredictable assembly errors, while machine assembly necessitates specific hardware equipment. Most VBTSs are assembled manually [20,27,28,41,47] or in hybrid form [35,37,56,63], with a limited number being fully assembled by machines [10,11].

#### Assembly workflow

The assembly workflow consists of serial assembly and parallel assembly. Serial assembly involves combining components in a specific sequential order, typically determined by structural design or manufacturing requirements. For example, DTac [27,28] consists of a transparent layer, a translucent layer, and a black layer, thereby requiring a fixed assembly order due to its stacked construction. In contrast, parallel assembly allows components to be assembled simultaneously, as seen in Shimonomura et al.’s [7] and Sato et al.’s sensors [34], which achieves this due to a simple structure. Some sensors will be assembled in a hybrid serial-parallel way; for example, the skin, marker, and base assemblies of Ward-Cherrier et al.’s [35], Lepora et al.’s [37], and Fan et al.’s sensors [56] can be combined in parallel via 3D print, but the internal elastomers have to be injected serially after the lens has been attached to the prints. Although parallel assembly is more efficient, it poses challenges for required complex processes and intricate structures. The majority of VBTSs are fabricated using serial assembly [20,23,47,49,62,75], while a minority employ a hybrid of serial and parallel assembly [35,37,56]. Purely parallel assembly is rarely used [10,11] due to obvious difficulty.

#### Assembly mechanism

The assembly mechanism consists of physical assembly and chemical assembly. Physical assembly relies solely on the interaction of hardware structures. For instance, Lin et al.’s [27,28], Zhang et al.’s [62], and Sferrazza and D’Andrea’s sensors [44] involve casting silicone into the base mold, thereby directly assembling the elastomer with the base. Chemical assembly utilizes the properties of chemical reagents, such as material compatibility and adhesion. For example, coating pigments are often mixed with silicone to enhance adhesion with the elastomer [20,21,60,61], and lenses are frequently attached to the base using adhesives [35,56]. Physical and chemical assemblies offer the benefits of processing convenience, adaptability for modular designs, and durability of product performance respectively based on the specific design; thereby, most VBTSs are produced through a hybrid of physical and chemical assembly [23,28,35,37,47,49], with only a few being made entirely through the physical [7,10] or chemical assembly [65].

**Fig. 2.**
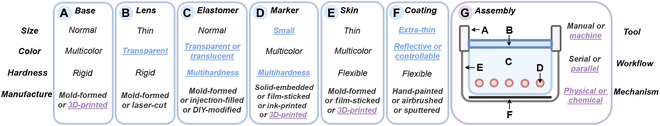
Representative attributes of a VBTS. (A) (Base) The external supporting structure. (B) (Lens) The transparent medium for camera imaging. (C) (Elastomer) The flexible main body. (D) (Marker) The physical medium for visualizing tactile information. (E) (Skin) The external layer for shading or protection. (F) (Coating) The functional layer for contact feature mapping. (G) (Assembly) The method of installing the aforementioned sub-components, involving various tools, workflows, and mechanisms.

In this section, we review the commonly used methods in the design and creation of VBTSs, whose representative attributes are summarized in Fig. 2. Each VBTS has several attributes with distinct characteristics, such as size, color, hardness, and manufacturing methods. This complexity corresponds to a wide range of structural variations, placing high demands on subsequent manufacturing and assembly processes. The complexities in process, time, and quality are difficult to address due to the limitations of traditional manufacturing methods. In Fan et al.’s paper [11], a monolithic manufacturing technique is proposed that has the potential to become a standard method to simplify the design and creation of VBTSs. Its feasibility was evaluated by using C-Sight, which relies on IMM. In this work, the CrystalTac family was designed as a series of sensors with different sensing mechanisms to demonstrate the adaptability of rapid monolithic manufacturing.

## Methods

Based on the details of monolithic manufacturing elaborated by Fan et al. [11], this work focuses on the design and creation of the CrystalTac family. The first step involves discussing the manufacturing feasibility of CrystalTac-type sensors based on different sensing mechanisms through monolithic manufacturing, followed by the overall design of the CrystalTac family.

### Manufacturing feasibility of different sensing-mechanism-based CrystalTac sensors

From Table 1 and Fig. 2, it is evident that different sensing-mechanism-based VBTSs exhibit certain tendencies in terms of sub-components, each associated with specific attributes and corresponding manufacturing methods. In detail, the choice of sensing mechanism directly determines the type of sub-component, while the specific sensor design based on that mechanism influences the attributes of each sub-component. The most striking issue is the significant variation in size, color, and hardness requirements for the various sub-components. Here, we discuss which sensing mechanism could be applied to CrystalTac-type sensors in terms of technological and practical feasibility.

#### Technological feasibility

As utilized in monolithic manufacturing, PolyJet printing (PP) [76] functions similarly to inkjet printing by spraying thousands of photopolymer droplets rather than ink. This process employs UV light to cure and construct parts in a layer-by-layer fashion. The combination of inkjet and photopolymerization technologies provides PP with 2 significant advantages:

• High printing quality: Due to the small size of the ejected resin droplets, impressive micron-level spatial resolution can be achieved in both the horizontal *XY* direction and the vertical *Z* direction. This level of precision is essential for achieving high print quality, characterized by fine resolution and a superior surface finish.
• Multimaterial printing: By integrating multiple print heads, PP easily achieves multimaterial printing with consistent print quality, whose printing capabilities encompass a wide variety of material properties. In addition to multicolor printing, it can also produce flexible materials with varying properties.

**Fig. 3.**
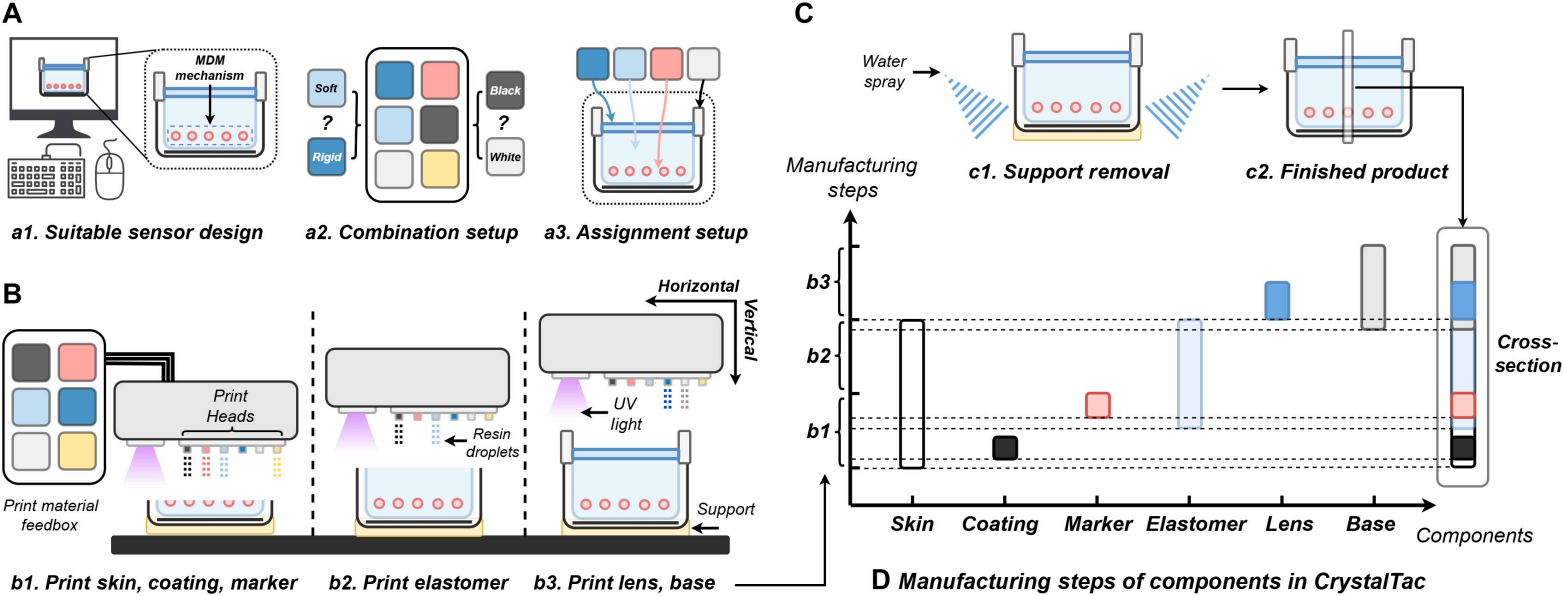
(A) The practical feasibility of CrystalTac depends on the suitable sensor design and the setup limitations of the print materials. (B) Rapid monolithic manufacturing integrates component fabrication and assembly into a single process. (C) The finished CrystalTac is ready for use after removing support structures, through either water spray or manual tools. (D) CrystalTac can realize complicated structural designs.

These 2 advantages have considerable significance in manufacturing different sensing-mechanism-based CrystalTac sensors with a complicated structure, as shown in Fig. 3B to D. Referring to the typical print materials listed in Fan et al.’s paper [11], Vero series (VB/VW/VC) (https://www.stratasys.com/en/materials/materials-catalog/polyjet-materials/vero/), Agilus30 series (AB/AW/AC) (https://www.stratasys.com/en/materials/materials-catalog/polyjet-materials/agilus30/), and support materials (https://www.stratasys.com/en/materials/materials-catalog/polyjet-materials/polyjet-support-materials/), are suitable selections for the skin, marker, elastomer, lens, and base of CrystalTac. These materials undergo a complete photopolymerization process during printing, resulting in nontoxic, and biocompatible final products. Therefore, CrystalTac sensors ensure a biologically friendly design. In addition, all printing materials are stored in sealed cartridges within the printer, allowing for precise dosing and efficient monitoring of usage, which helps minimize material waste during the production process. In contrast, traditional methods, such as mold casting, often leave significant material residue in preparation containers, leading to unnecessary wastage. The efficient use of materials in monolithic manufacturing thus contributes positively to sustainability. There have been several existing VBTSs fabricated using the abovementioned printing materials, such as those by Scharff et al. [13], Ward-Cherrier et al. [35], and Fan et al. [56]; thereby, the CrystalTac family of the same designed sensing mechanism should have a performance similar to that of those research studies in terms of sensor performance determined by the material, such as sensitivity, resolution, durability, and multimodality capability. However, due to the lack of print materials containing metal powder, reflective or controllable coatings are currently unavailable, rendering some IMM VBTSs based on these coatings, such as GelSight, not yet feasible for CrystalTac. It can be easily solved by developing appropriate printing materials in the future.

#### Practical feasibility

As displayed in Fig. 3A, the practical feasibility depends on the suitable design of each sensor, as well as the combination and assignment setups of loaded print materials.

• Suitable sensor design: As illustrated in Fig. 3A (a1), the structural design of CrystalTac must be based on a defined mechanism. For example, MDM CrystalTac requires the marker pattern to be embedded in the correct position, MFM CrystalTac often requires minimal occlusion within the imaging path, and IMM CrystalTac may need a multilayer stacked structure. An irrational structural design can fail to achieve the desired sensing mechanism. However, the efficiency and flexibility of monolithic manufacturing can help researchers rapidly implement hardware iterations of a prototype design by controlling variables.
• Combination setup of print material: It should be noted that the print materials loaded in the feedbox are limited, and the number of print heads is also fixed by the printer version, as shown in Fig. 3A (a2). Therefore, it is necessary to consider the actual material loading situation of the current printer, especially with multimechanism fusion designs that require a more complex range of print materials. In general, the minimum criteria to be fulfilled include at least 3 print materials, of which AC, VW/VB, and support materials must be loaded. If there are spare print heads available, priority should be given to loading AW/AB or VC. Such strategies can maximize the design options for CrystalTac with limited material combinations.
• Assignment setup of print material: After determining the sensor design, it is necessary to assign the appropriate material to each sub-component based on different attributes, as shown in Fig. 3A (a3), considering various factors such as color, hardness, and shape. For example, MFM CrystalTac requires high transparency and low distortion levels in the skin, while MDM CrystalTac requires effective shading. It should be emphasized that although the number of print materials loaded is limited, their properties can be adjusted through fusion with each other. For instance, by mixing flexible Agilus30 materials into the rigid Vero series, materials with intermediate hardness can be obtained, while VeroVivid (https://www.stratasys.com/en/materials/materials-catalog/polyjet-materials/verovivid/) can be combined with a wide range of materials to achieve rich colors. These characteristics can be adjusted by changing the mixing ratio, and the entire process is automated by the printer, greatly enhancing design flexibility and ensuring production quality.

The above analysis demonstrates that rapid monolithic manufacturing can provide sufficient capability to create the CrystalTac family with different sensing mechanisms but given certain prerequisites. As seen in Section S5.1, taking into account the optimization for key sub-components of CrystalTac, a thickness range of 2 to 3 mm is optimal for printed lenses, offering a balance between high structural strength and satisfactory imaging performance. Similarly, the suitable height for the printed elastomer should range between 2 and 5 mm to balance image quality and hardness. Compared with traditional manufacturing methods, such as mold-casting manufacturing, rapid monolithic manufacturing based on multimaterial 3D printing provides the CrystalTac sensor significant advantages in manufacturing with complex structures for 2 specific reasons: (a) Modification of the internal substructure affects only the design part of the sensor (Fig. 3A) but not the creation part (Fig. 3B), so as long as a suitable computer-aided design (CAD) model can be modeled and within the performance range of the PolyJet machine, the customization of the CrystalTac sensor may not affect the actual production. (b) During the creation of the sensor, both the manufacture and assemble processes are carried out in parallel (Fig. 3D), which reduces assembly errors and saves a lot of assembly time. A demonstration of these advantages is provided in Section S5.4.

### Overall design of the CrystalTac family

**Fig. 4.**
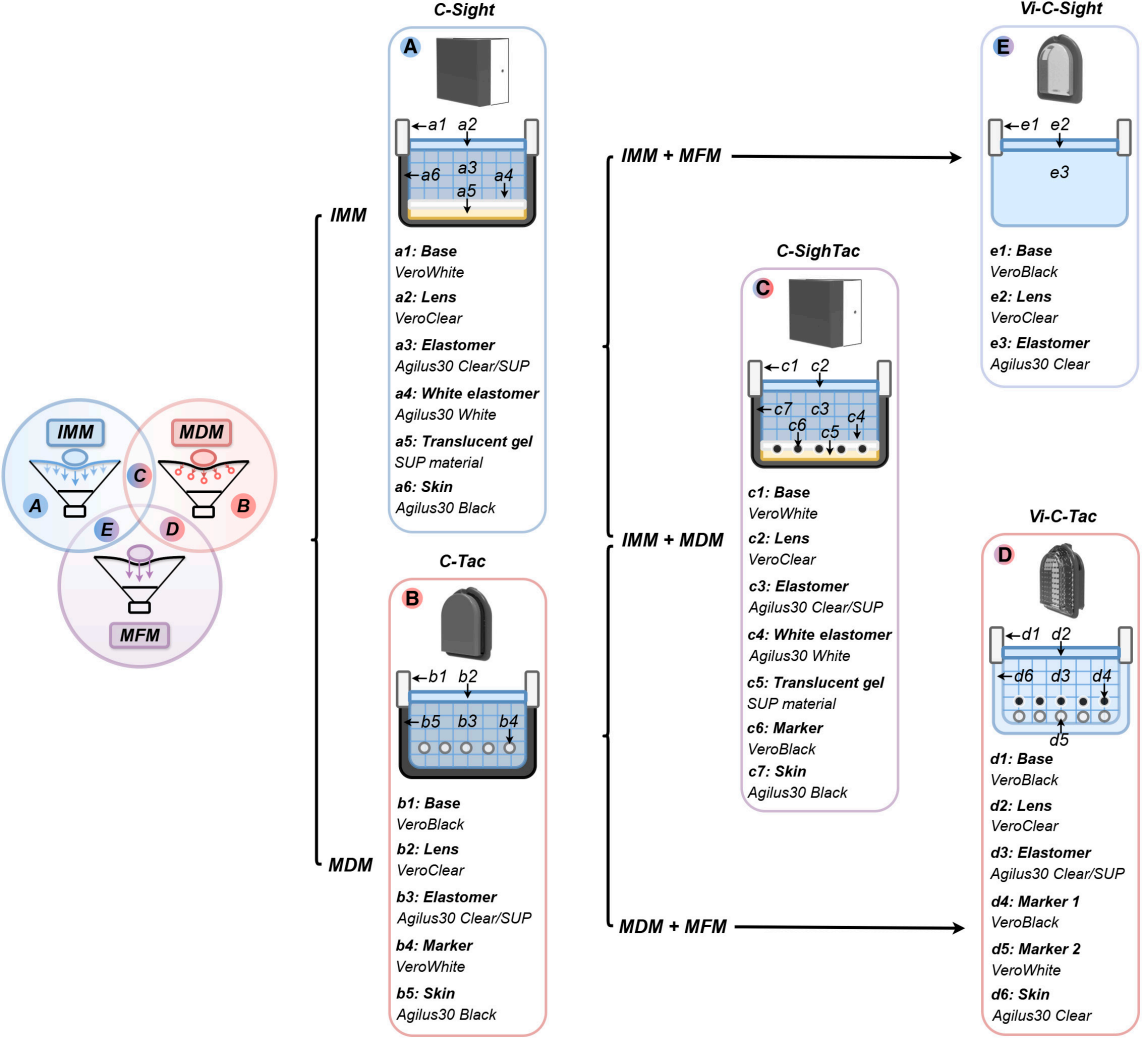
The family tree of CrystalTac comprises 5 major branches, each proposed with a different tactile sensing mechanism. (A) C-Sight, IMM mechanism. (B) C-Tac, MDM mechanism. (C) C-SighTac, IMM + MDM mechanism. (D) Vi-C-Tac, MDM + MFM mechanism. (E) Vi-C-Sight, IMM + MFM mechanism. The diagram for each design illustrates its unique internal structure and material assignment.

The CrystalTac family design is introduced here, whose family tree is illustrated in Fig. 4. According to Fig. 1A, the CrystalTac family is categorized into 5 branches, C-Sight using the IMM mechanism, C-Tac using the MDM mechanism, C-SighTac using the IMM + MDM mechanism, Vi-C-Sight using the IMM + MFM mechanism, and Vi-C-Tac using the MDM + MFM mechanism. It should be emphasized that these 5 CrystalTac sensors are proposed not to provide a final design version but rather to validate their potential to implement various tactile sensing mechanisms.

#### Contact module

• C-Sight: To realize the IMM mechanism, C-Sight has been designed, as described by Fan et al. [11], inspired by DTac [27,28]. The sectional diagram in Fig. 4A shows that within the gap between C-Sight’s outer skin (a6) and the clear elastomer (a3), 2 additional components are present: a translucent layer (a5) composed of support material and a pure white layer (a4) made of Agilus30 White. The deformation resulting from external contact alters the distance between the black skin and the white layer. Where the distance is shortened, the pixel intensity appears darker, aiding in inferring the contact depth.
• C-Tac: To achieve the MDM mechanism, C-Tac can be embedded with 2D and 2.5D markers through different material assignments as shown in Fig. 4B, such as rigid Vero and flexible Agilus30 series. The distinction between them lies in the ability to provide pseudo-depth information, achieved either through shape deformation or variations in morphological structure. Monolithic manufacturing allows for customization of both the hardness properties and the morphological geometry of the markers. As shown in Section S5.2, 3 kinds of marker patterns can be employed for C-Tac, including multilayer markers, Voronoi markers, and coordinate markers, each possessing distinct characteristics compared to dot markers.
• C-SighTac: As illustrated in Fig. 4C, C-SighTac is developed based on C-Sight and C-Tac to achieve the IMM + MDM mechanism. This approach involves the structural framework of C-Sight and marker designs from C-Tac into appropriate positions. Similar to marker-enhanced GelSight [47], markers may enable C-SighTac to be more sensitive to dynamic features, such as force.
• Vi-C-Tac: Inspired by Finger Vision [55] and ViTacTip [56], Vi-C-Tac uses transparent skin to replace the opaque one of C-Tac as displayed in Fig. 4D, aiming to realize the MDM + MFM mechanism. The internally embedded markers provide greater dynamic sensitivity to physical interaction while retaining the multimodality sensing capability through the clear skin.
• Vi-C-Sight: Vi-C-Sight utilizes the IMM + MFM mechanism, inspired by Shimonomura et al.’s sensor [7]. Using the TIR principle, variations in the gradient of the elastomer surface upon the contacted object alter the trajectory of internally reflected light beams. This causes the contact texture to be mapped onto the transparent elastomer surface, resulting in imaging brightness change while still retaining background visual information. As seen in Fig. 4E, Vi-C-Sight uses pure Agilus30 Clear as the elastomer material to leverage this mechanism rather than a multilayer grid structure.

#### Vision and illumination modules

To demonstrate CrystalTac’s customization flexibility for prototype designs and its adaptability for modifying mature designs, 2 bases with different vision and illumination modules are applied to the 5 proposed sensors. The first customized base is designed for C-Sight and C-SighTac, characterized by a square body with 6 internal white illumination sources, as detailed by Fan et al. [11]. The other base is from the well-known Digit [22], which features a small black curved housing with an RGB light source. It is a popular commercial VBTS product due to its suitability for mounting on robot hands and its quick-change contact module design. So we used it as the base for C-Tac, Vi-C-Tac, and Vi-C-Sight. A description of these 2 bases is provided in Section S5.3.

## Results

In this section, we designed several experiments to evaluate the CrystalTac family across a variety of task targets. Initially, the performance of CrystalTac with different sensing mechanisms was verified through 3 functional tasks. As C-Sight has been tested on the tactile reconstruction task [11], it, along with the similar C-SighTac, was not further evaluated. Instead, the focus was on C-Tac, Vi-C-Tac, and Vi-C-Sight, which were selected for object recognition, object and texture hybrid recognition, and see-through-skin exploration, respectively. It is noted that our purpose in introducing the experiment here is not to highlight how outstanding the performance of the CrystalTac family is, as the exact performance metrics depend on the iterative optimization of the sensor structure, as well as the usage scenario of the task. Instead, we prefer to provide a real-life example to verify that rapid monolithic manufacturing can help the CrystalTac family achieve a specific design of the sensing mechanism. It also encourages other researchers to utilize this technique in their own sensor creation for rapid design iteration and flexible customization. In other words, the CrystalTac family is not in competition with other VBTSs that can belong to the CrystalTac family if they follow a similar design and are manufactured using rapid monolithic manufacturing. Thereby, the CrystalTac family is more of a collection of VBTS products that utilize rapid monolithic manufacturing. Additionally, 2 further evaluations were conducted on the manufacturing cost and customization flexibility of CrystalTac.

### Object recognition

**Fig. 5.**
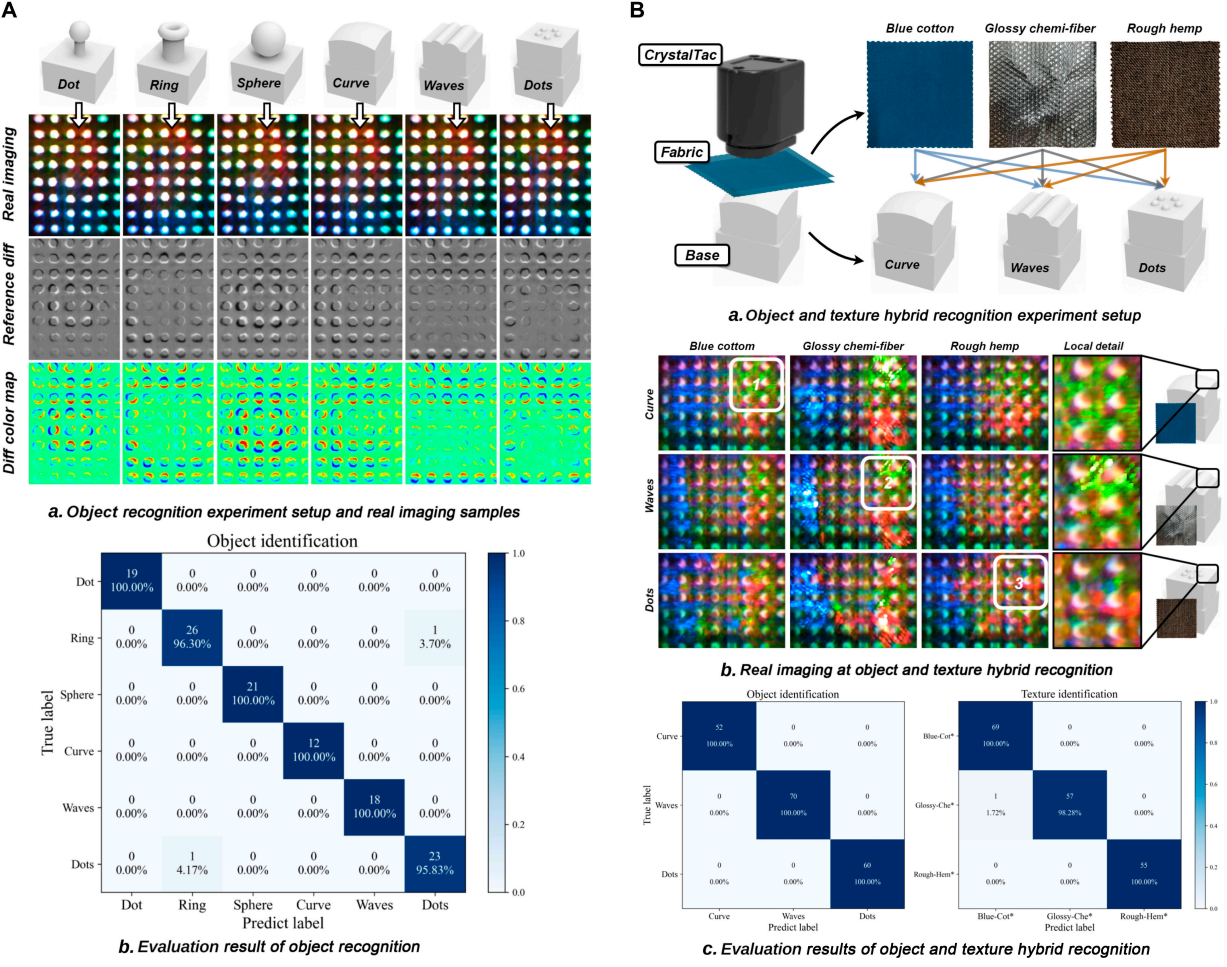
(A) Setup for object recognition using C-Tac with single-layer dot markers. (a) The marker pattern distributions change with different contact objects. (b) The marker features are effective for precise object identification. (B) Setup for the object and texture hybrid recognition using Vi-C-Tac with double-layer markers. (a) By covering the fabric, the sensing of both visual and tactile features can be simultaneously evaluated. (b) Fine textures are clearly visualized, while object shape mapping relies primarily on double-layer marker patterns. (c) Both objects and textures can be identified using Vi-C-Tac. Blue-Cot*, blue cotton; Glossy-Che*, glossy chemi-fiber; Rough-Hem*, rough hemp.

C-Tac with single-layer dot markers was used to achieve the object recognition task. This sensor features a black skin to prevent external light interference and a white marker pattern that is sensitive to mapping skin deformation. As illustrated in Fig. 5A(a), 6 print parts were selected, namely, a dot, a ring, a sphere, a curve, waves, and multiple dots. The difference between the reference image and the image after contact can indicate both the location and extent of deformation. For a marker that has been displaced or deformed, its original position is indicated in red, while the final position is indicated in blue; the absence of these colors suggests that the marker remained stationary. For instance, when the hollow ring is in contact, only the peripheral markers of C-Tac are displaced, whereas the centrally located markers are the most displaced when for the sphere. Because of the limited density of the marker pattern, it is hard to distinguish “waves” and “dots” from marker images. The IMM-based VBTS works better in tactile reconstruction, such as C-Sight [11].

Subsequently, a total of 1,200 images were collected, with 200 images per class, to train the object recognition models. A Densenet121 [77] model was employed for data inference, and the evaluation results are summarized in Fig. 5A(b). With a test accuracy of 98.3471%, the trained model correctly identified 119 out of 121 images in the test set, demonstrating C-Tac’s capability to characterize tactile information from physical contact based on the MDM mechanism.

### Object and texture hybrid recognition

To evaluate the MDM + MFM mechanism provided by Vi-C-Tac, a hybrid recognition experiment was designed, with the setup illustrated in Fig. 5B(a). The experiment involved 3 different fabrics—blue cotton, glossy chemical fiber, and rough hemp—as well as 3 print parts: curve, waves, and dots. By wrapping the fabric around the objects, visual information alone was insufficient to distinguish the print objects, although it allowed for capturing the fine texture of the fabric. Thereby, the markers were utilized to capture tactile features, enabling the analysis of the shape of the underlying parts. A Vi-C-Tac system with double-layer dot markers was employed. A total of 9 permutations between these fabrics and parts were tested, as shown in Fig. 5B(b). The enlarged local details revealed distinct textures: the cotton fabric displayed clear horizontal grain and the chemical fiber exhibited point textures with strong reflective properties, while the features of hemp were less distinct due to its rough characteristics. The double-layer markers were discernible within the view, with nonoverlapping parts of the markers differentiated by color contrast.

For each permutation case, 200 images were collected, resulting in a total of 1,800 images for model training. By adding 2 separate head structures following the output of Densenet121, 2 classifiers for object recognition and texture recognition were implemented in a decoupled manner. The test results, shown in Fig. 5B(c), indicate identification accuracies of 100% (182/182) for object recognition and 99.45% (181/182) for texture recognition. Both visual and tactile features were successfully captured by Vi-C-Tac, thanks to the MDM + MFM mechanism.

### See-through-skin exploration

A Vi-C-Sight was employed to conduct see-through-skin exploration, utilizing its IMM + MFM mechanism. The elastomer, made up entirely of Agilus30 Clear, functions as a light-conductive plate [7], enabling the TIR effect to achieve the fusion of proximity-tactile sensing. Deformed areas are differentiated in brightness from undeformed areas, while other regions remain highly transparent, thereby facilitating the see-through-skin capability.

**Fig. 6.**
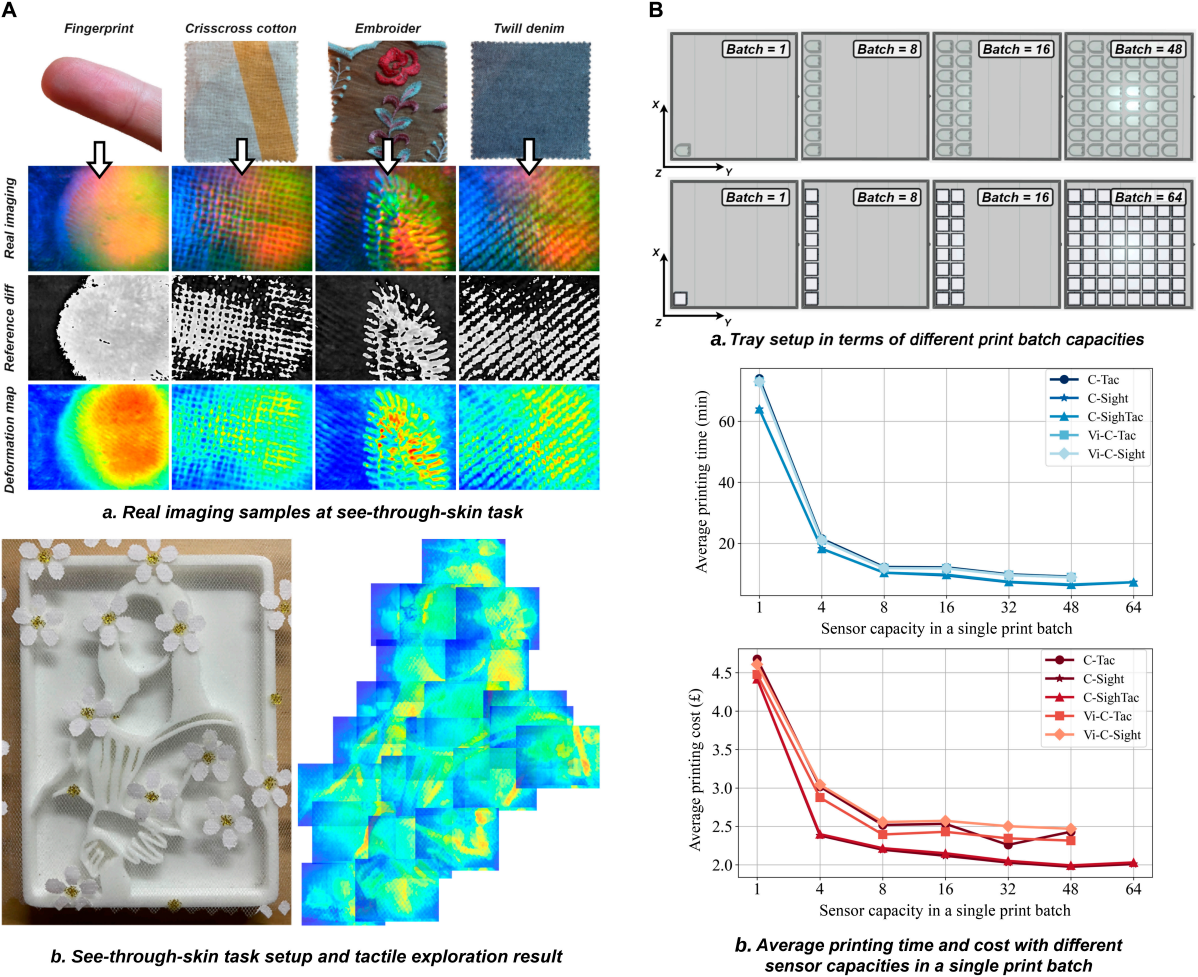
(A) Setup for the see-through-skin exploration using Vi-C-Sight. (a) Pure Agilus30 Clear provides a clear view of the visual–tactile fusion feature. (b) Both the fine texture of the fabric and the geometric shape of the rigid base can be captured. (B) Evaluation of CrystalTac’s manufacturing cost. (a) The maximum capacity for CrystalTac using the Digit base is 48 units, compared to 64 units using a customized base. (b) As the print batch increases, both the manufacturing time and cost decrease gradually until a stable level is reached.

As shown in Fig. 6A(a), fine textures on the objects’ surfaces are clearly captured. For instance, fingerprints are visible without interference from other marker patterns, allowing Vi-C-Sight to produce such clear imaging that the entire outline of the finger is recognizable against a darker background. A distinct line of demarcation marks the contact areas where textures are mapped onto the elastomer surface, akin to glass. The fabric fibers can embed themselves into the elastomer during contact, enhancing the TIR effect. This effect can be observed in the geometry of crisscross cotton, the fiber orientation of twill denim, and the level of embroidery deformation, which can all be mapped to variations in pixel intensity. Unlike Vi-C-Tac, the MFM mechanism of Vi-C-Sight inherently couples visual and tactile information through changes in the luminance of the pixels, similar to TIRgel [62]. In Fig. 6A(b), an embossed print base with a human figure was wrapped in a skeleton fabric. Vi-C-Sight was used to explore the surface of these combined parts and generate an exploration map. Due to the sparse nature of the fibers, the fabric was not as visually distinctive as the opaque printed adhesive pattern also present. However, the figure pattern of the embossed print base beneath, where the 2 overlap, is visible in the reddish color of the embossed areas, indicating a higher degree of deformation and a pronounced fabric texture in the immediate vicinity.

Here, we summarize the sensor performance with different sensing mechanisms in Table 2: (a) The C-Tac sensor can successfully identify bases with various shapes using an MDM-based design, offering stability against external interference due to the protection of the opaque skin. However, it lacks the ability to perceive fine textures upon contact because of the limitation in marker density. (b) Vi-C-Tac addresses this issue through the MDM + MFM mechanism, allowing it to capture the visual features of the contact surface, although it fails to obtain contact depth. (c) In contrast, Vi-C-Sight can detect the depth of contact textures relying on the IMM + MFM mechanism but cannot capture dynamic tactile features, which is an advantage of the MDM mechanism.

**Table 2.** Comparison of sensor performance with different sensing mechanisms

Sensor	Mechanism	Task for case studies	Advantages	Disadvantages
C-Tac	MDM	Object recognition	Insensitive to external noise	Inability to perceive fine texture
Vi-C-Tac	MDM + MFM	Object and texture hybrid recognition	Vision aid in fine texture perception	Sensitive to noise, lack of depth information of texture
Vi-C-Sight	IMM + MFM	See-through-skin exploration	Able to perceive depth information of texture	Sensitive to noise, inability to perceive dynamic tactile feature

It is evident that the selection of an appropriate sensing mechanism plays a decisive role in the overall performance of a VBTS. At the outset of VBTS design, we should specify the relevant performance indices based on predefined usage scenarios and tasks, choosing the most suitable mechanism by considering the characteristics of different sensing methods. In other words, there is no universally optimal performance among different sensing mechanisms, only the one most suitable for the current task. Once the most appropriate sensing mechanism is finalized, rapid monolithic manufacturing can implement the VBTS design in the most convenient and expeditious manner, facilitating researchers to experiment and iterate with ease.

### Manufacturing cost evaluation of the CrystalTac family

An evaluation of manufacturing costs similar to that by Fan et al. [11] was applied to the CrystalTac family, with the results summarized in Table 3. For C-Tac with single-layer dot markers, Vi-C-Tac with double-layer dot markers, and Vi-C-Sight, all of which utilize a Digit base, C-Tac is slightly larger in size and volume due to the additional layer of opaque skin. C-Sight and C-SighTac, which use a customized base, have volumes similar to those of the others due to their rectangular shape. This difference in shape is reflected in the unit print speed (T/V) and unit material consumption cost (C/V), as C-Tac/Vi-C-Tac/Vi-C-Sight possesses complex curved surfaces with overhangs, necessitating additional support materials. This requirement reduces the unit print speed and increases the unit material consumption cost. For instance, the T/V and C/V for C-Tac/Vi-C-Tac/Vi-C-Sight are approximately 11.5 min/cm^3^ and £0.73/cm^3^, respectively, which are higher compared to those of C-Sight/C-SighTac, with around 10 min/cm^3^ and £0.685/cm^3^, respectively. Therefore, it can be concluded that the closer the shape of a CrystalTac component is to a rectangle, the more efficient it is to print through monolithic manufacturing, thereby lowering the manufacturing cost.

**Table 3.** Manufacturing costs of different sensors within the CrystalTac family. Cost excludes 20% VAT.

Sensor	Size *X*/*Y*/*Z* (mm)	Volume (cm^3^)	AG (g)	VR (g)	DG (g)	Sup (g)	Time (min)	Cost (£)	*T*/*V* (min/cm^3^)	*C*/*V* (£/cm^3^)
C-Tac	34 × 27 × 16.5	6.538	9	12	3	10	74	4.678	11.318	0.716
C-Sight	26.5 × 26.5 × 13.5	6.446	10	10	3	8	64	4.418	9.929	0.685
C-SighTac	26.5 × 26.5 × 13.5	6.446	10	10	3	8	64	4.418	9.929	0.685
Vi-C-Tac	34 × 27 × 16.15	6.208	8	12	3	10	73	4.478	11.759	0.721
Vi-C-Sight	34 × 27 × 16.15	6.208	9	12	3	9	73	4.608	11.759	0.742

VAT, value-added tax; AG, Agilus30 series; VR, Vero series; DG, DraftGrey; Sup, support; *T*/*V*, unit print speed; *C*/*V*, unit material consumption cost

As shown in Fig. 6B, further evaluations have been conducted concerning different sensor capacities within a single print batch. For all 5 types of CrystalTac sensors, the maximum number along the *X* direction on the print tray is 8, while it varies in the *Y* direction—6 for C-Tac/Vi-C-Tac/Vi-C-Sight and 8 for C-Sight/C-SighTac—resulting in maximum capacities of 48 and 64, respectively. As the print batch capacity approaches these maximum values, both the average printing time and cost decrease sharply until reaching a batch capacity of 8, after which they stabilize at a slightly lower level. This pattern aligns with the conclusions obtained by Fan et al. [10]. The underlying reason is that when the capacity exceeds 8, additional motion overheads are required in the *Y* direction for the extra columns. For example, in the case of C-Tac, when the batch capacity is 48, the average print time and cost are only 9.08 min and £2.43, representing decreases of 87.73% and 48.05% from 74 min and £4.678, respectively, when the batch capacity is 1.

### Customized flexibility evaluation of the CrystalTac family

As discussed, customized flexibility is an essential metric for reducing design complexity. With monolithic manufacturing, as long as the computer-aided design model of CrystalTac is designed, it can be manufactured directly without any additional processes. This capability allows for the realization of many complex designs to meet the requirements of specific tasks.

#### Customized internal structure

**Fig. 7.**
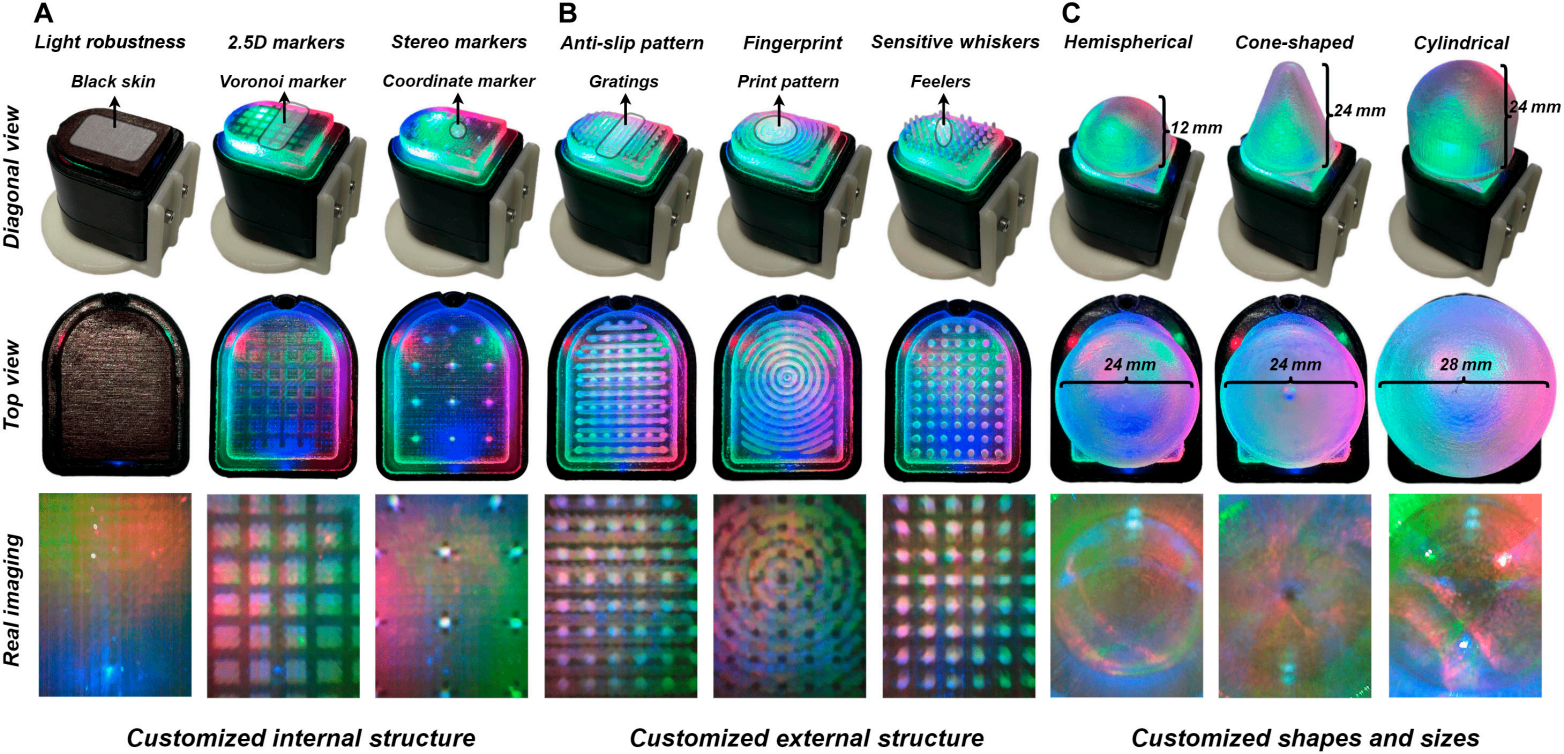
Customized flexibility test of CrystalTac. (A) The internal structure of CrystalTac can be flexibly customized. (B) The external accessories are free to add. (C) The scales and shapes are also customizable.

As shown in Fig. 7A, for designs that require only tactile information, light robustness is enhanced by using Agilus30 Black for the outer skin, which blocks external ambient light. Additionally, the 2.5D markers made of soft material can deform to provide richer tactile information. In contrast, markers made of rigid material maintain their structure while still moving with deformation, enabling the creation of more complex geometries. Further design examples of novel markers and complicated structures can be seen in Sections S5.3 and S5.4.

#### Customized external structure

The external structure plays a crucial role in tactile sensing, particularly in dynamic interactions. As shown in Fig. 7B, the anti-slip texture can increase friction between the sensor surface and external objects, effectively reducing undesirable sliding and providing a solid foundation for robust gripping tasks. This texture also protects the contact module body by being wear resistant, thus extending the service life and reducing usage costs. Furthermore, whiskers can enhance the sensitivity of built-in markers. By leveraging the principle of micro-leverage effect, tactile information such as shear or pressure received at the external end is magnified at the internal end.

#### Customized shapes and sizes

The ability to modify the size and shape of the contact module is also a key feature. Compared to the traditional flat shape, spherical and cylindrical surfaces perform better in in-hand manipulation tasks since they can obtain a greater sensing area. However, these surfaces are more difficult to manufacture, especially internal lenses, which often require complicated processes that challenge traditional manufacturing methods. As shown in Fig. 7C, a hemispherical sample and 2 additional samples with cone-shaped and cylindrical structures were manufactured. Their built-in lenses are consistent with the overall shapes, with elastomers all 2 mm thick, matching the lens thickness.

## Conclusions

In this work, we designed the CrystalTac family, comprising 5 branches each with a unique sensing mechanism design. To effectively bridge the gap between VBTS design and creation phases, rapid monolithic manufacturing is proposed based on our previous research, demonstrating its potential to become a universal manufacturing technology of VBTSs. To resolve the challenge of “what kind of VBTS can be designed for monolithic manufacturing”, we investigated the rule within the design and creation of VBTSs and we summarized the sensing mechanisms and manufacturing and assembly methods of typical VBTSs. Based on the concluded result, 5 types of CrystalTac sensors were fabricated using rapid monolithic manufacturing: C-Tac, C-Sight, C-SighTac, Vi-C-Tac, and Vi-C-Sight. Subsequent functional experiments demonstrated that the CrystalTac sensors perform well and meet their design targets with unique tactile sensing mechanisms. Additionally, rapid monolithic manufacturing has led to significant improvements in design flexibility and manufacturing costs, often constrained by conventional methods of manufacturing and assembly.

The CrystalTac family can be regarded as an initial template, with no strict parameter limitations for each sensor detail, thereby encouraging the community to view it as a foundation for further development. The contribution of this work lies in demonstrating the capability of rapid monolithic manufacturing to produce VBTSs with various tactile sensing mechanisms, providing confidence and inspiration to other researchers in the tactile robotics field. In future work, we aim to advance the new CrystalTac series by enhancing the capabilities of monolithic manufacturing in terms of production quality and efficiency, as well as multimaterial printing for VBTSs. These technologies can be seamlessly integrated with tactile sensory enhancements in dexterous hands to perform tasks such as human–computer interaction or dexterous manipulation.

## Data Availability

Please contact the authors to obtain data, including the design details of the CrystalTac family.

## Supplementary Materials

Supplementary 1 Supplementary Text Figs. S8 to S12 Ref. [78–80]
