# Supplementary material for: CrystalTac: Vision-Based Tactile Sensor Family Fabricated via Rapid Monolithic Manufacturing
Source: Cyborg Bionic Syst. 2025 Apr 10;6:0231. doi: 10.34133/cbsystems.0231 (PMC11982672; doi:10.34133/cbsystems.0231)
Supplement: Supplementary 1 — Supplementary Text Figs. S8 to S12 Ref. [78–80] [file cbsystems.0231.f1.docx]

## Supplementary Materials

### 5.1 Optimisation on Sub-components Manufacturing of CrystalTac

Several extended experiments have been conducted to explore the optimal range of sub-component attributes in CrystalTac, including the lens and elastomer. Considering the coupled influence of the lens and elastomer on the mechanical and optical performance, we chose a decoupled approach in their optimisation, evaluating each part individually without considering interference with each other. Firstly, the specific performance of the VBTS depends on the design details and task requirements, which need several iterations for modification, so a reasonable range of choices is enough for optimised initialisation. Further, the structural dimensions of VBTS tend to be small in size, almost in millimeter level of contact module thickness, and therefore lead to less interference between parts.


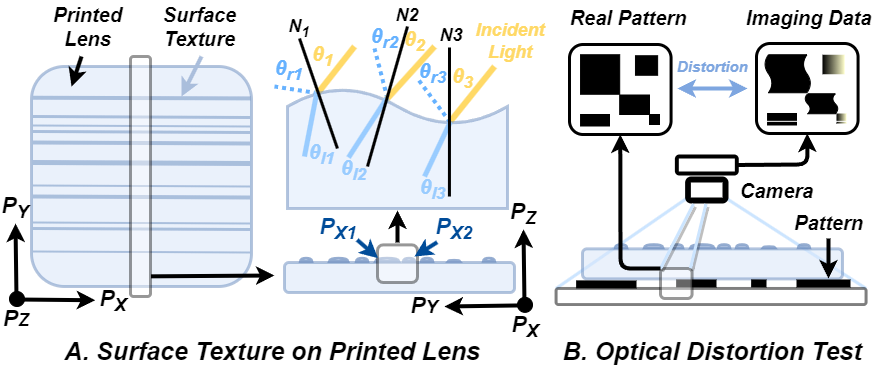


Figure 8: A: Horizontal textures on the printed lens surface. Adjacent textures form a wavy pattern, complicating the refraction and reflection of incident light. B: These surface textures can result in unpredictable distortion between the actual pattern and the camera imaging.

#### 5.1.1 Optimisation for CrystalTac Lens Manufacturing

VeroClear is highly effective for manufacturing lens components, being a nearly colourless material that exhibits dimensional stability. Its properties are comparable to those of Polymethyl methacrylate (PMMA), commonly known as acrylic. Its upgraded version, VeroUltraClear, offers 95% light transmission and improves upon VeroClear with higher clarity, transparency, and a lower yellow index. We produced a batch of lens samples using VeroClear, which demonstrated good transparency. However, there were still strip-shaped textures present on the surface of the printed lenses, as depicted in Fig. 8(A).

A potential cause of these textures could be the presence of incoherent clogging in the tiny nozzles inside the print head, which prevents the formation of a continuous inkjet along the Y-direction so that axial movement along X results in the alternating superimposition of different widths in Px. For instance, two adjacent movements of working nozzles in the print head, Px1 and Px2, separated by $\Delta$Py, produce a deviation $\Delta$Pz in the vertical height Pz-an uneven texture will be created between the Px1 and Px2 trajectories. These textures complicate the processes of refraction and reflection as incident light passes through the material. Assuming that the normal incidence angles of three parallel beams on the lens surface are N1, N2, and N3, with angles of incidence $\theta$1, $\theta$2, and $\theta$3, respectively, the angles of reflection for their reflected light will be $\theta$r1, $\theta$r2, and $\theta$r3. It is evident that these reflected beams are no longer parallel. Similarly, for light refraction, which follows Snell’s law as shown in Eq. 1, the refractive indices $N_{lens}/N_{air}$ of the two media (lens and air) are greater than 1. Therefore, the refraction angles $\theta$l1, $\theta$l2, and $\theta$l3 will be slightly smaller than $\theta$1, $\theta$2, and $\theta$3, and these refracted beams will also not be parallel to each other.

$\frac{\sin\theta}{\sin\theta l}=\frac{N_{lens}}{N_{air}}$ (1)


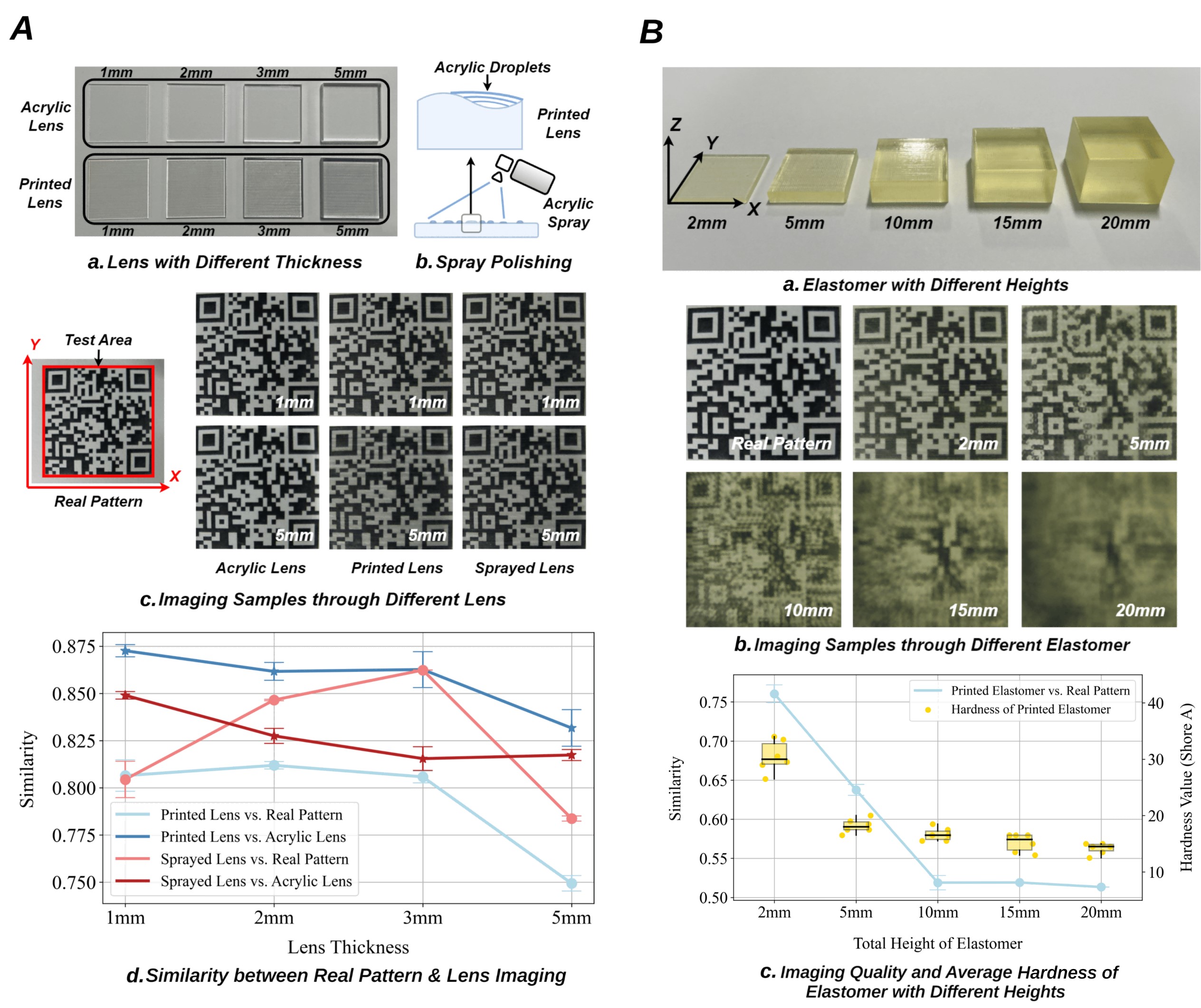


Figure 9: A: Optimisation of printed lens for CrystalTac. (a) Test samples of printed lenses and acrylic lenses were compared; (b) The acrylic spray can enhance the glossy of the printed lens; (c) Real imaging for evaluating lens quality; (d) Imaging quality test of the lenses was conducted, with the error value indicating the difference between similarity values in X/Y directions. B: Optimisation of printed elastomer for CrystalTac. (a) Test samples of printed elastomer with different heights were compared; (b) Real imaging for evaluating elastomer quality; (c) Trend of imaging quality and hardness with elastomer thickness change.

When applied to CrystalTac, the aforementioned phenomenon of printed lenses leads to two problems. Firstly, internal illumination can cause irregular reflections on the lens surface. Secondly, images captured through the lens may exhibit optical distortion, as demonstrated in Fig. 8(B). Both issues significantly impair the quality of the tactile data acquired by CrystalTac. The first problem can be addressed by optimising the design of the illumination system. The second issue, however, differs from common optical lens distortions such as barrel distortion, pincushion distortion, and mustache distortion, which can be corrected using distortion parameters derived from chessboard pattern calibration. This optical distortion arises from the randomly shaped surface of the lens, leading to unpredictable outcomes that cannot be corrected through simple parameter adjustments. This issue is evidently a consequence of the operating principles of pp, which, despite having a surface finish superior to most other 3D printing technology. To avoid redundant post-processes such as mechanical polishing, two methods have been tested to determine if the imaging quality of the printed lens surface can be improved:

• **Adjusting Printing Thickness**:

As shown in Fig. 9(A.a), four lenses with different thicknesses were printed: 1mm, 2mm, 3mm, and 5mm. All lenses have planar dimensions of 30mm x 30mm. For the control group, four additional acrylic lenses of the same size were fabricated using laser cutting. As indicated in Fig. 9(A.c), by placing a QR code pattern beneath the lenses, a camera positioned above can capture the pattern’s imaging through the lenses. To quantitatively analyse the optical distortion level, the Structural Similarity Index Measure (SSIM) was introduced as the metric to evaluate image similarity. This approach assesses similarity by considering a combination of factors, including image luminance, contrast, and structure. To avoid the influence of surface texture direction, each printed lens was placed over the QR pattern along both the X and Y directions, capturing two image samples. From Fig. 9(A.d), the similarity between images taken through printed lenses and acrylic lenses is around 86% for thicknesses less than 3mm. However, as thickness increases to 5mm, similarity decreases to 83%. This decrease is primarily attributed to the lower light transmittance of VeroClear compared to acrylic, with excessive thickness resulting in darker images through the printed lenses. The highest similarity score, approximately 87.5%, is attained with lenses 1mm thick, suggesting that some distortion may be due to surface texture. Similarly, when comparing the similarity of the printed lens to the actual pattern, there is an overall decrease of about 7%, indicating that acrylic lenses also contribute to some degree of distortion.

• **Applying Acrylic Spray**:

As introduced in Stratasys official guidance of finish process[[1]](#footnote-1), spray on a coat of clear lacquer or polyurethane can give luster to the VeroClear model and protect its finish. Following this, we apply acrylic spray can fill the uneven texture on the lens surface with liquid droplets as illustrated in Fig 9(A.b). Once cured, these droplets enhance the overall finish of the lens, offering a more convenient and efficient alternative to physical polishing with sanding equipment. In our test, all four printed lenses were placed together, and a canister of acrylic spray was applied from a distance of 1m for approximately 2 seconds. After curing for an hour in ventilated conditions, the liquid acrylic layer on the surface was ready for testing, with all settings identical to the previous tests. As shown in Fig. 9(A.d), the similarity between images taken through sprayed lenses and acrylic lenses decreased overall by 3-5% compared to images from printed lenses without post-processing. However, the similarity of the sprayed lens to the real pattern improved, reaching a maximum of 86% with 3mm thick lenses. The improvement in the impact of surface texture attributed to the spray is indicated by the reduced disparity in similarity values across the X and Y directions for lenses of all thicknesses.

In summary, altering the printing thickness and applying acrylic spray both impact the imaging quality of printed lenses. Considering various factors, a thickness range of 2-3mm is optimal for printed lenses, providing a balance between structural strength and satisfactory imaging performance. Building on this foundation, the application of acrylic spray improves the surface finish of the printed lens.

#### 5.1.2 Optimisation for CrystalTac Elastomer Manufacturing

Most VBTSs require a transparent, silicone-like elastomer as the core material to facilitate technologies such as IMM, MDM, MFM, and multi-mechanism fusion. As introduced in [64], a mechanical characterisation is applied to evaluate the stress-strain curve and life circle of 3D-printed skin. For our work, the printed elastomer is mainly based on commercial 3D printed materials, Agilus30[[2]](#footnote-2), whose tensile strength is around 2.4-3.1MPa, elongation at break is around 220-270%, tensile tear resistance is 5-7Kg/cm. For example, Agilus30 Clear, due to its transparent and flexible texture, is suitable for replacing silicone in elastomer or skin of the CrystalTac family. However, the Shore hardness of pure Agilus30 is 30-35A, which is too hard for designs requiring a softer elastomer. For instance, GelSight typically uses a Shore hardness range of 5-20A [15]. By incorporating the multi-layer grid structure proposed in MagicTip [10], the hardness of the printed elastomer can be further reduced by adjusting the inner layer amount. To assess the impact of this structure on imaging quality, five test samples were manufactured. These samples shared the same X/Y dimensions of 30mm x 30mm for the elastomer, but varied in height: 2mm, 5mm, 10mm, 15mm, and 20mm, as shown in Fig. 9(B.a). The skin layer’s thickness was set to 0.5mm to balance a soft texture with stable strength. The same QR code pattern and SSIM metric used for evaluating the imaging quality of printed lenses, as depicted in Fig. 9(A.b), were employed.

As displayed in Fig. 9(B.b), when the height of the printed elastomer increases, the imaging quality decreases in two ways. Firstly, the image tone gradually becomes darker and tends toward a darker yellow. This is primarily due to the support material filled in the internal core, which has lower light transmission than the Agilus30 material. However, these tests were conducted without external illumination. In practical VBTS applications, illumination modules are typically present, and the emitted light can mitigate the impact of material coloration Secondly, the imaging quality deteriorates, leading to a gradual loss of detail in the pattern beneath. This trend is confirmed by Fig. 9(B.c), where the similarity between the printed elastomer imaging and the real pattern is 76% at a height of 2mm but gradually drops to around 52% when the height exceeds 10mm.

We also observed that the overall height can affect hardness when it is less than 5mm, even if the outer skin thickness remains constant. This is mainly because reducing the elastomer height decreases the number of layers in the embedded grids, and too few layers limit the range of deformation possible in the support core. According to our test results, when the elastomer thickness is between 2-5mm, the number of layers embedded within the grid should be approximately 1-4. Consequently, the hardness gradually increases from Shore 20A to 30A–the hardness of pure Agilus30–suggesting that as the number of layers decreases, the printed elastomer hardens until it effectively becomes pure Agilus30. When the thickness exceeds 5mm, the hardness stabilises at around 15A, indicating that the additional deformation capacity provided by the multi-layer grid structure reaches a plateau once the number of layers exceeds four.

For CrystalTac, to optimise the imaging quality and elasticity of the printed elastomer with the multi-layer grid structure, the suitable thickness ranges of such elastomer are 2mm to 5mm, where thinner thickness leads to better transparency and thicker thickness results in the softer hardness. This rule provides valuable design references for other users on their prototype design through rapid monolithic manufacturing.

### 5.2 Marker Design for CrystalTac Family

How to choose the appropriate marker pattern is the design focus of MDM-type VBTS. As discussed in [36], different marker geometries each have unique advantages and disadvantages, and the selection of markers should be made according to the preset performance of VBTS and the estimated usage scenarios. At the same time, different marker geometries will also have an impact on the production method, and rapid monolithic manufacturing can greatly alleviate the problem of difficult-to-manufacture markers, especially those that have a 3D spatial design of and need to be aligned along complex surfaces in a strict and precise position. Below we introduce several marker patterns that can be manufactured by rapid monolithic manufacturing and discuss their respective performance:

• **Dot Marker**: Dot markers are among the most common patterns in VBTSs [38, 18, 55]. Regarding shape, dot markers can include both cylinders with slender thicknesses and spheres with symmetrical dimensions. The overall marker array distribution can be categorised into either uniform or random spatial arrangements. Furthermore, dot markers can be transformed into pin-shaped markers by integrating them with the skin, a feasibility demonstrated in [39, 17, 40]. As shown in Fig. 10(A), if a rigid material such as Vero is used for dot marker manufacturing, the printed pattern will exhibit properties of a 2D marker. However, if flexible materials like Agilus30 or Tango are used, the markers have the potential to deform into various shapes. This deformation is an additional feature, categorising them within the realm of 2.5D markers. The precise pattern of the markers is dictated by the design specifications established during CAD modelling, which involves considerations of both the number and density distribution of markers, allowing for either a uniform or a random distribution.

• **Double-layer Marker**: When the elastomer contacts the object, the extent of elastic deformation decreases along the longitudinal axis. The double marker approach utilises this inhomogeneity by capturing the difference in distribution between the upper and lower pattern layers to map tactile information [13]. As shown in Fig. 10(B), this contrasting disparity typically enhances specific dynamic tactile characteristics, such as shear motion, compared to a single-layer marker configuration. The fabrication process for double-layer markers is similar to that of dot markers, with the primary distinction being the inclusion of an additional marker layer in the CAD modelling. Moreover, the construction principles of spatial structures comprising more than two layers remain consistent. By increasing the number of layers, the extreme case is that the different layers of the marker transition from a discrete state of separation to a continuous state of interconnection, resembling a top-to-bottom cylinder. By controlling the gradient distribution of colours, it should be possible to provide a more fine-grained mapping of the tactile information. All of the above variants can be easily implemented through monolithic manufacturing.


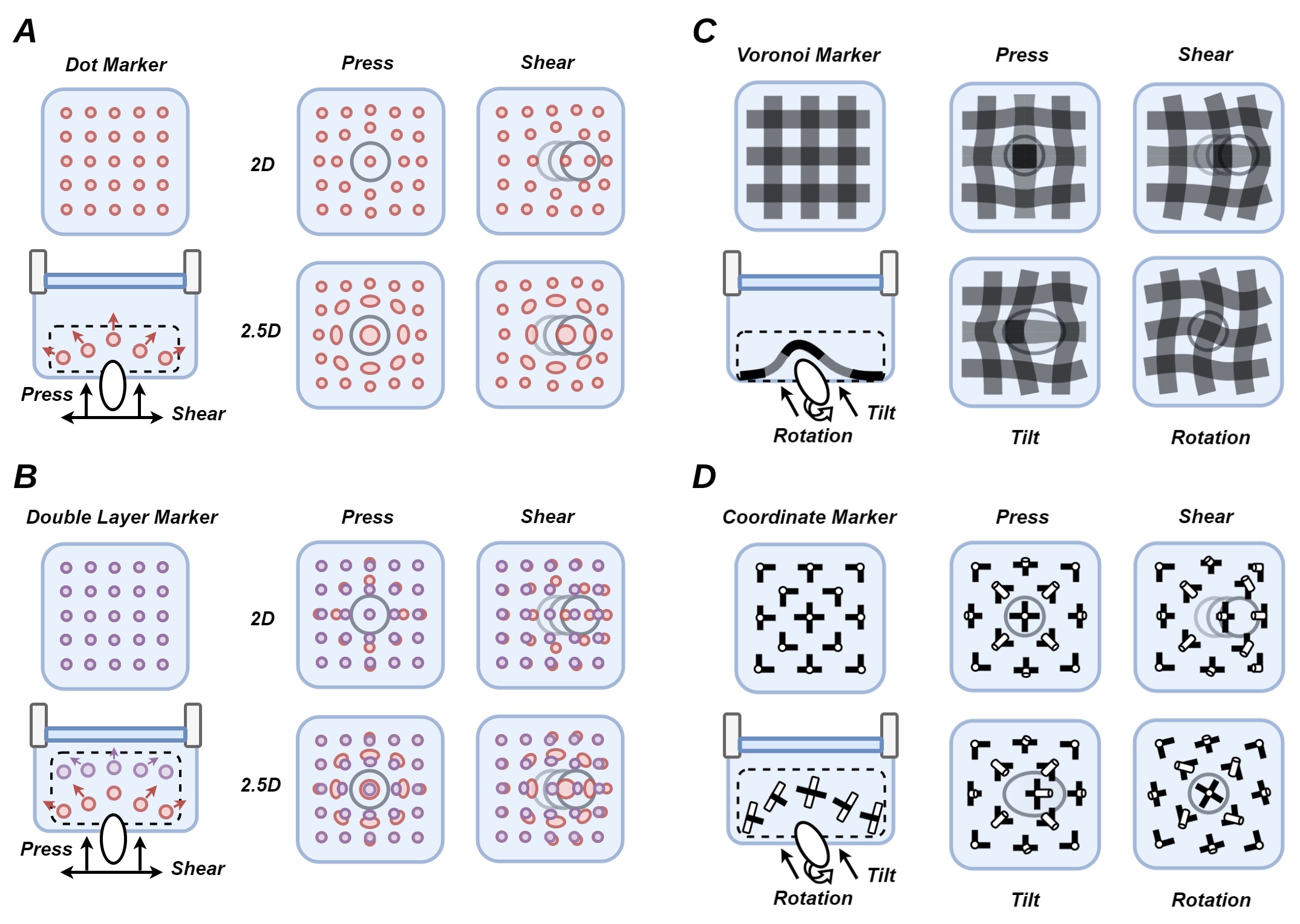


Figure 10: Marker design for CrystalTac. A: Schematic of dot marker. The pattern of dot markers is sensitive to translations along the contact plane. Compared to 2D dot markers, 2.5D dot markers can change their shapes to provide more features about depth. B: Double-layer markers enrich tactile features through changes between the upper and lower-layer patterns. 2.5D double-layer markers extend this capability from geometric distribution to shape variety. C: The Voronoi marker represents a geometric advancement over the 2.5D dot marker. These regularly arranged geometric tessellations use variations in the continuity of corner points, edges, colour, and area of each inlay to map tactile features. D: Each coordinate marker is designed as a gyroscope-like 3D pointer structure, allowing each marker to individually map 6D pose information. This design improves the tactile representation, aiding in force estimation or dynamic motion tracking.

• **Voronoi Marker**: Similar to the lattice pattern in [77], the Voronoi marker is designed with a continuous pattern distribution in a square layout as shown in Fig. 10(C). Each edge of such a square marker can be viewed as a collection of dot markers, thus improving spatial resolution compared to the standard dot marker, as investigated in continuous marker pattern (CMP) [48]. Using monolithic manufacturing, Voronoi markers can be seamlessly integrated into the fabrication of the skin or embedded at any specific spatial layer within the elastomer, according to a customised design. However, the marker design must adhere to geometric principles, where each tessellation cell of the Voronoi pattern should be a polygon, such as a triangle, square, or hexagon.

• **Coordinate Marker**: Most marker patterns share two characteristics: each marker typically has a regular spatial shape, such as a sphere or dot, and multiple markers are grouped into an array to map force, shear, or other contact information. These characteristics imply that a single marker plays a limited role in the overall array due to the relatively low marker density. Through monolithic manufacturing, we introduce the coordinate marker, as illustrated in Fig. 10(d). Each individual marker in the array can independently infer the 6D pose of its localised elastomer. This capability stems from the distinct design of each marker, akin to a three-dimensional pointer within a gyroscope, making it sensitive to both rotational and translational movements. Consequently, markers characterised by complex spatial structures, as opposed to regular shapes, can be classified as stereo markers(including proposed coordinate markers). To our knowledge, the design and creation of such stereo markers are achievable exclusively through monolithic manufacturing.


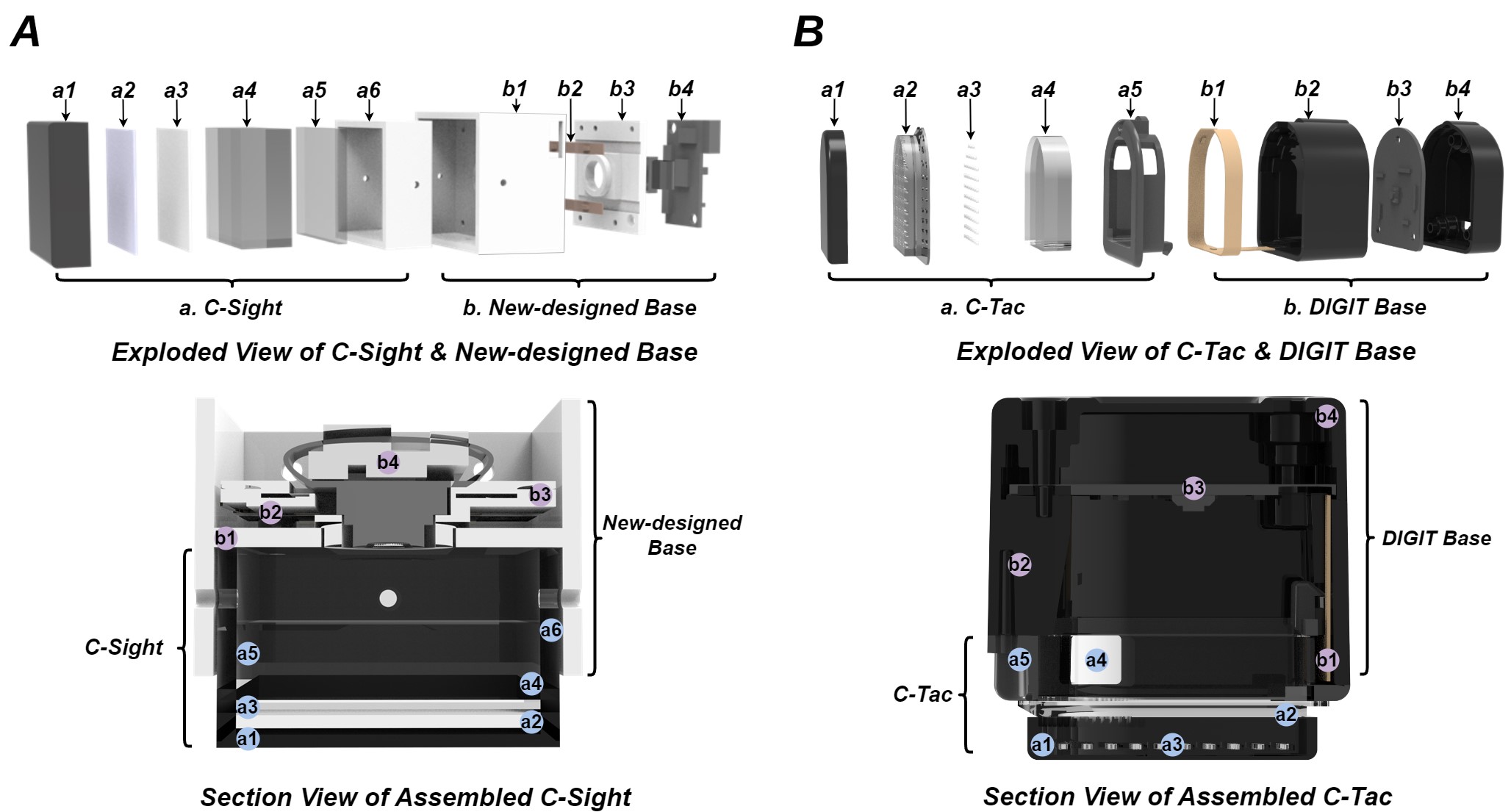


Figure 11: Base design for proposed CrystalTac, the exploded view, and section view of assembled C-Sight and C-Tac are introduced here for reference. A: New-designed base [11] for C-Sight and C-SighTac, (a1)black skin; (a2)translucent gel; (a3)white elastomer; (a4)clear elastomer; (a5)lens; (a6)base of contact module; (b1)mounting base; (b2)LED; (b3)camera base; (b4)camera. B: Commercial Digit base for C-Tac, Vi-C-Tac and Vi-C-Sight, (a1)black skin; (a2)clear elastomer; (a3)marker; (a4)lens; (a5)base of contact module; (b1)LED; (b2)mounting base; (b3)camera; (b4)Bottom base.

### 5.3 Vision and Illumination Design for CrystalTac Family

The proposed rapid monolithic manufacturing focuses on the fabrication of the contact module in VBTS, while the remaining two modules - illumination module and camera module - are briefly introduced here. To highlight the applicability of the CrystalTac family, two bases were designed, including a square base with a completely customized design, and a direct use of the commercial Digit base, as shown in Fig. 11(A)/(B). For various VBTS development scenarios, such as prototyping from scratch or iteratively upgrading an existing design, the CrystalTac family, based on rapid monolithic manufacturing, meets the needs.

### 5.4 Printing Sample of Customised CraystalTac

In order to highlight the advantages of rapid monolithic manufacturing, a sample was created that combines a number of designs from the CrystalTac family, as shown in Fig. 12. It is clear that such a complex multi-material coupled structure would face significant challenges if fabricated using conventional methods.


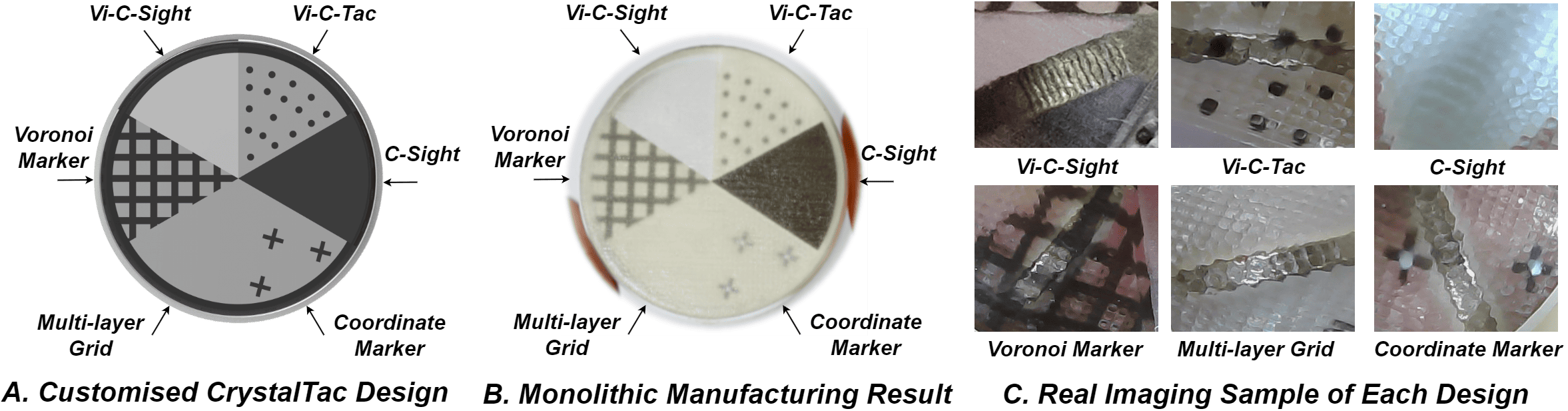


Figure 12: Rapid monolithic manufacturing provides CrystalTac family superior design flexibility and creation efficiency. A: The CAD model of customised CrytalTac example which integrates six different designs, which is hard to be fabricated by traditional methods. B: The real product of such complicated design through monolithic manufacturing. C: The images of different customised designs with a coin as stimuli.

1. https://support.stratasys.com/en/Applications/Finishing-Processes [↑](#footnote-ref-1)
2. https://www.stratasys.com/en/materials/materials-catalog/polyjet-materials/agilus30/ [↑](#footnote-ref-2)
